# Dispersal Capacity Rather Than Shared Environmental Constraints Determines Taxon‐Specific Demographic Dynamics in an Alpine Lake Network

**DOI:** 10.1111/mec.70173

**Published:** 2025-11-13

**Authors:** Joaquín Ortego, Eduardo Franco‐Fuentes, Susana Pallarés, José A. Carbonell, Daniel Caballero‐Fernández, Pedro Abellán

**Affiliations:** ^1^ Department of Ecology and Evolution Estación Biológica de Doñana, EBD‐CSIC Seville Spain; ^2^ Department of Zoology, Faculty of Biology University of Seville Seville Spain; ^3^ Department of Ecology and Hydrology, Faculty of Biology University of Murcia Murcia Spain

**Keywords:** alpine lakes, demography, diving beetles, genetic diversity, landscape genetics, macroinvertebrate aquatic communities

## Abstract

Networks of alpine lakes and ponds support unique assemblages of aquatic organisms and provide an ideal biogeographical setting for studying the evolutionary, ecological and demographic outcomes of population fragmentation. In this study, we integrate genomic, morphological and community‐level data within a comparative multi‐taxon framework to investigate genetic connectivity, demographic trajectories and eco‐evolutionary dynamics in four diving beetles (Coleoptera: Dytiscidae) representative of the macroinvertebrate assemblages inhabiting high altitude lakes in the Sierra Nevada massif, southeastern Iberia. Although the focal taxa share similar ecological requirements, primarily occupy lentic habitats and disperse by flight, our results reveal substantial heterogeneity in their demographic responses to the naturally fragmented distribution of alpine lakes. Taxa with higher wing loading exhibited stronger genetic differentiation among populations, probably due to their reduced capacity to disperse across the direct geographic distances separating lakes. Populations located at the range periphery tended to exhibit lower genetic diversity than central populations in all taxa. Demographic reconstructions showed a general decline in effective population size from the last glacial maximum (LGM) to the present. However, some populations of genetically more structured taxa went through brief bottlenecks that coincided with periods of warmer climate and lower lake levels, as inferred from local paleoclimatic reconstructions. Finally, the composition of macroinvertebrate assemblages (α‐diversity and β‐diversity) was not associated with intra‐specific genetic diversity or differentiation, suggesting that species‐level demographic trajectories and community‐level dynamics are decoupled. Our findings indicate that interspecific differences in dispersal capacity outweigh shared environmental constraints in determining the contrasting demographic trajectories of the studied taxa. Collectively, these results emphasise the importance of multi‐taxon approaches for understanding the dynamics of species assemblages in alpine ecosystems that are highly vulnerable to climate warming.

## 1 Introduction

Alpine ecosystems—high‐elevation habitats above the treeline—cover less than 3% of the Earth's land surface outside Antarctica (Testolin et al. 2020). Despite their limited extent, they harbour a disproportionately high number of species compared to other regions (Steinbauer et al. 2016; Rahbek et al. 2019). High‐mountain habitats, particularly at lower latitudes, often form ‘sky islands’ embedded within an extensive matrix of lowland environments with warmer climates that are inhospitable to cold‐adapted organisms (Flantua et al. 2020). Consequently, most alpine species occur in highly isolated populations, often representing local endemics restricted to one or a few mountain ranges (Steinbauer et al. 2016). This severe fragmentation becomes particularly relevant in the context of ongoing climate warming, whose effects—amplified in alpine ecosystems (IPCC 2022; e.g., Alps, Central Europe: Gobiet et al. 2014; Sierra Nevada, southeastern Iberia: Jiménez‐Moreno et al. 2023) – are accelerating the contraction of alpine habitats and driving both local and global extinctions of numerous cold‐adapted taxa (Wilson et al. 2005; Dirnbock et al. 2011). The rich biodiversity and high sensitivity of alpine ecosystems to climate warming make them not only important biodiversity hotspots of great conservation concern, but also sentinels for detecting early‐warning signals of the detrimental impacts of global change on biodiversity (e.g., Wilson et al. 2005; Vanneste et al. 2017).

A distinctive feature of alpine landscapes is the presence of networks of lakes and ponds that support unique assemblages of aquatic organisms (Lamouille‐Hébert et al. 2024). High‐mountain lakes can be considered ‘islands within sky islands’, offering an ideal biogeographical setting for studying the evolutionary, ecological and demographic consequences of extreme population fragmentation in a highly vulnerable ecosystem (Lamouille‐Hébert et al. 2024). Climate change is predicted to increase water temperature, shorten hydroperiods and reduce connectivity in alpine lake networks (e.g., Carlson et al. 2020; Jiménez‐Moreno et al. 2023), directly threatening the persistence of alpine aquatic communities (Weckström et al. 2016; Moser et al. 2019). Thus, understanding genetic connectivity among alpine lake populations is key to evaluating their resilience to environmental disturbances, including the potential for re‐colonisation following stochastic extinction events (e.g., due to lake desiccation in extreme years). Unfortunately, our knowledge of metapopulation dynamics in alpine lake networks remains limited (Lamouille‐Hébert et al. 2024). Only a handful of studies have analysed spatial patterns of genetic diversity and structure at a landscape scale in alpine lake networks, mostly relying on genetic markers (e.g., AFLP, mtDNA gene fragments) with limited resolution for demographic inference or estimating contemporary gene flow (Čiamporová‐Zaťovičová and Čiampor 2017; Macko et al. 2025; Ventura et al. 2014).

Co‐distributed species with similar ecological requirements may respond very differently to habitat fragmentation, depending on life history traits that determine dispersal capacity, reproductive rates and effective population sizes. Consequently, the synergic effects of habitat fragmentation and climate warming are unlikely to impact all species equally. Species' responses to spatiotemporal landscape and environmental heterogeneity will depend on their intrinsic capacity to adapt—via evolutionary change and/or phenotypic plasticity—and their ability to disperse and re‐colonise increasingly fragmented and unstable habitats (Lamouille‐Hébert et al. 2024; Pallarés et al. 2020). For these reasons, multi‐species frameworks aimed at understanding the processes governing population genetic connectivity (e.g., dispersal capacity, landscape configuration) and demographic trajectories (e.g., habitat stability, past environmental changes) in alpine aquatic organisms are crucial for gaining insights into the factors that shape and maintain species assemblages, for assessing community‐level responses to habitat fragmentation and climate warming and, ultimately for identifying taxa most vulnerable to their detrimental effects (Čiamporová‐Zaťovičová and Čiampor 2017; Lamouille‐Hébert et al. 2024).

In this study, we integrate genomic, morphological and community‐level data within a comparative multi‐taxon framework to investigate population genetic connectivity, demographic trajectories and eco‐evolutionary dynamics in four species of diving beetles (Coleoptera: Dytiscidae) representative of the macroinvertebrate assemblages inhabiting alpine and subalpine lakes of the Sierra Nevada massif, southeastern Spain. Sierra Nevada, the highest mountain range in the Iberian Peninsula, has been identified as a super‐biodiversity hotspot, characterised by exceptional species richness and high levels of local endemism, resulting from its unique geographic, geological and climatic history (Arroyo et al. 2022). Macroinvertebrate communities in the network of alpine ponds and lakes of Sierra Nevada are dominated by diving beetles (Abellán et al. 2022), which constitute an excellent model system to investigate the genetic consequences of habitat fragmentation and the demographic responses of alpine biotas to past and ongoing global change (e.g., Pallarés et al. 2020). Their dominance in these ecosystems (Abellán et al. 2022), contrasting dispersal abilities (Arribas, Velasco, et al. 2012; Hjalmarsson et al. 2015) and high sensitivity to environmental fluctuations (Arribas, Abellán, et al. 2012; Pallarés et al. 2020) make them particularly suitable for addressing questions about genetic connectivity, adaptation and resilience in fragmented alpine landscapes highly vulnerable to climate warming (Bilton et al. 2019).

Specifically, in this study, we first (i) quantified genetic structure and spatial patterns of differentiation, testing the hypothesis that taxa with greater dispersal ability—as inferred from morphometric proxies—exhibit increased gene flow and population connectivity. Second, we (ii) applied a spatially explicit landscape genetics framework to evaluate the relative roles of geographical and topographic distances, as well as environmental dissimilarity (i.e., elevation), in shaping genetic structure across the four focal taxa. Third, (iii) we tested the hypothesis that population genetic diversity is positively associated with both the geographic centrality of populations and lake area, as expected if demographic performance decreases toward distributional limits and small habitat patches support lower effective population sizes (*N*_e_) (Lira‐Noriega and Manthey 2014). Fourth, we (iv) used a coalescent‐based approach to infer historical changes in *N*_e_ and to evaluate whether genetically more fragmented taxa have experienced more heterogeneous demographic trajectories and population bottlenecks associated with deteriorating environmental conditions (i.e., warmer temperatures, reduced lake levels), as inferred from local paleoclimate reconstructions (Jiménez‐Moreno et al. 2023; López‐Blanco et al. 2024). Finally, (v) we tested the hypothesis that species richness (α‐diversity) and turnover (β‐diversity) of the macroinvertebrate community are correlated with within‐species genetic diversity and differentiation, respectively, as expected from the parallelism between key eco‐evolutionary processes—selection/species sorting, gene flow/dispersal, genetic drift/ecological drift and mutation/speciation—operating at both intra‐specific and community levels (Vellend and Geber 2005; Lamy et al. 2017; Govaert et al. 2021).

## 2 Materials and Methods

### 2.1 Study Area and Sampling

The study area includes subalpine and alpine ponds and lakes from the Sierra Nevada mountain range, southeastern Iberia, belonging to the oro‐Mediterranean (1900–2900 m a.s.l.) and cryoro‐Mediterranean (above 2900 m.a.s.l.) bioclimatic belts (Rivas‐Martínez 1990). The network of alpine lakes and ponds in Sierra Nevada formed after the glacier retreat following the last glacial cycle (Castillo Martín 2009). These lakes are subject to extreme environmental conditions (prolonged ice cover, high UV radiation, oligotrophic conditions) and host simplified aquatic macroinvertebrate communities of cold‐adapted species dominated by water beetles, including widely distributed species and local endemics (Abellán et al. 2022). Our sampling focused on four diving beetles (Coleoptera: Dytiscidae), including the Sierra Nevada endemics *Agabus nevadensis* Lindbeg, 1939 and *Hydroporus sabaudus sierranevadensis* Shaverdo, 2004, the western Mediterranean *Boreonectes ibericus* (Dutton & Angus, 2007) and the western Palearctic *Hydroporus marginatus* (Duftschmid, 1805). These four species are the most representative and common diving beetles inhabiting the system of high altitude lakes in Sierra Nevada (Abellán et al. 2022), in which they dominate the macroinvertebrate community and frequently co‐occur. For each focal species, we sampled between 8 and 10 populations covering their respective distributions within the Sierra Nevada mountain range. We used an aquatic hand net to collect 8–12 specimens per locality. Specimens were preserved in 96% ethanol and stored at −20°C until needed for genomic analyses. In a previous study, we found that some populations of the alpine *A. nevadensis* hybridise with the elevation generalist *A. bipustulatus* Linnaeus, 1767 (Pallarés et al. 2024). For this reason, we excluded from the dataset all individuals with hybrid/admixed ancestry identified by Bayesian clustering analyses in structure (*q*‐value < 0.99). For details on these analyses, see Pallarés et al. (2024) and Section 2.3. Further details on sampling sites and the number of genotyped individuals for each species are presented in Table S1.

### 2.2 Genomic Library Preparation and Processing

We extracted and purified DNA from each specimen using NucleoSpin Tissue kits (Macherey‐Nagel, Düren, Germany). We processed DNA into different genomic libraries using the double‐digestion restriction‐fragment‐based procedure (ddRAD‐seq) described in Peterson et al. (2012). In brief, we digested DNA with the restriction enzymes MseI and EcoRI (New England Biolabs, Ipswich, MA, USA) and ligated Illumina adapters containing unique 7‐bp barcodes to the resulting fragments from each individual. We then pooled the ligation products, size‐selected fragments between 350 and 450 bp using a Pippin Prep machine (Sage Science, Beverly, MA, USA), amplified them by PCR for 12 cycles with the iProof High‐Fidelity DNA Polymerase (BIO‐RAD, Veenendaal, The Netherlands) and sequenced the libraries in single‐read, 201‐bp lanes on an Illumina NovaSeq 6000 platform. We used the different programs distributed as part of the stacks v. 2.66 pipeline (Rochette et al. 2019) to filter and assemble our sequences into de novo loci, call genotypes, calculate genetic diversity statistics and export input files for all downstream analyses. Unless otherwise indicated, for all downstream analyses, we exported only one random SNP per RAD locus (option *write‐random‐snp*) and retained loci that were represented in at least 75% of individuals (*R* = 0.75). For more details on genomic data filtering and assembling, see Methods S1.

### 2.3 Population Genetic Structure and Gene Flow

We quantified genetic structure and admixture across populations of each species using the Bayesian Markov chain Monte Carlo clustering method implemented in the program structure v. 2.3.3 (Pritchard et al. 2000). We ran structure analyses assuming correlated allele frequencies and admixture and without using prior population information. We conducted 15 independent runs for each value of *K* (from *K* = 1 to *K* = 10) to estimate the most likely number of genetic clusters with 200,000 MCMC cycles, following a burn‐in step of 100,000 iterations. We retained the ten runs having the highest likelihood for each value of *K* and determined the number of genetic clusters that best describes our data according to log probabilities of the data (LnPr(X|*K*); Pritchard et al. 2000) and the Δ*K* method (Evanno et al. 2005), as implemented in structure harvester (Earl and vonHoldt 2012). We used clumpp v. 1.1.2 and the Greedy algorithm to align multiple runs of structure for the same *K*‐value (Jakobsson and Rosenberg 2007) and distruct v. 1.1 (Rosenberg 2004) to visualise the individuals' probabilities of population membership in bar plots.

To complement the above, we performed principal component analyses (PCA) as implemented in the r v. 4.3.2 (R Core Team 2024) package ‘adegenet’ (Jombart 2008). Before running PCAs, we replaced missing data by the mean allele frequency of the corresponding locus estimated across all samples (Jombart 2008). We also calculated genetic differentiation between each pair of populations using the Weir & Cockerham weighted fixation index (*F*_ST_) (Weir and Cockerham 1984), as implemented in arlequin v. 3.5 (Excoffier and Lischer 2010). Statistical significance was determined using Fisher's exact tests with 10,000 permutations, and a false discovery rate (FDR) correction (5%, *q* < 0.05) was applied to account for multiple testing. Pair‐wise *F*_ST_ values were calculated only for populations with five or more genotyped individuals (Table S1), a sample size shown in previous ddRAD‐seq studies to provide reliable estimates of genetic differentiation (González‐Serna et al. 2020; e.g., Ortego et al. 2021).

Finally, we used the function *divMigrate* implemented in the r package ‘diveRsity’ v1.9.90 (Keenan et al. 2013) to examine directional relative migration between populations of each species (Sundqvist et al. 2016). We estimated gene flow as *Nm* (i.e., effective number of migrants) and performed 1000 bootstrap iterations to assess whether gene flow between each pair of populations was significantly asymmetric. We plotted the resulting matrix with the r package ‘qgraph’ (Epskamp et al. 2012), disregarding edges below 0.35.

### 2.4 Landscape Genetic Analyses

We applied a landscape genetic approach to test whether genetic differentiation between populations (pair‐wise *F*_ST_; calculated as indicated in Section 2.3) of each focal species (i.e., response distance matrix) was explained by the following explanatory variables (i.e., predictor distance matrices):

- Geographical distance: The geodesic distance between sampled populations was calculated using the r package ‘geodist’ (Padgham and Sumner 2025).
- Weighted topographic distance: We used a 30‐m resolution digital elevation model (DEM) from NASA's Shuttle Radar Topographic Mission (SRTM) (https://portal.opentopography.org/) to calculate weighted topographic distances between each pair of populations, as implemented in the r package ‘topodistance’ (Wang 2020). We calculated weighted topographic paths using the *topoWeightedDist* function, with a linear function to weigh the angle of aspect changes and an exponential function to weight the slope between cells, as recommended by Wang (2020). These topographic distances account for the additional overland distance covered by an organism due to elevation changes imposed by topographic relief and assume that the energetic cost to traverse a slope varies exponentially with the change in angle.
- Elevation dissimilarity: As an estimate of environmental dissimilarity, we calculated differences in elevation between each pair of populations based on Euclidean distances.

We analysed the data using multiple matrix regressions with randomisation (MMRR; Wang 2013). Because geographical and environmental distances are only expected to have a positive effect on the degree of genetic differentiation between populations, we employed one‐tailed hypothesis tests to evaluate the null hypothesis of no effect of independent variables on genetic differentiation (Ruxton and Neuhäuser 2010; e.g., Yannic et al. 2018). Geographical and weighted topographic distances were highly inter‐correlated across all taxa (*r* > 0.90, *p* < 0.001), whereas correlations between these two variables and elevation dissimilarity were non‐significant in all cases (*r* < 0.45, *p* > 0.05). Such high collinearity between geographical and weighted topographic distances could potentially lead to spurious relationships between predictors and the response variable. To account for this, we ran two sets of models, each including one of the two inter‐correlated variables (i.e., either geographical or weighted topographic distances), and selected the model providing the best fit to the data based on the adjusted coefficient of determination (*R* ^2^). Each set of models was initially constructed as a full model with all explanatory terms (i.e., elevation dissimilarity and either geographical or weighted topographic distances) included. The final model was then selected using a backward stepwise procedure, progressively removing non‐significant variables (starting with the least significant) until only significant terms remained. Finally, we tested the significance of excluded terms against the reduced model to confirm that no additional variable reached significance. This approach resulted in the minimal adequate model best explaining variability in the response variable, where only significant explanatory terms were retained (e.g., Ortego, García‐Navas, et al. 2015).

### 2.5 Population Genetic Diversity

We used the program *populations* from stacks (see Methods S1) to calculate Wright's inbreeding coefficient (*F*_IS_) and different estimates of population genetic diversity, including observed heterozygosity (*H*_O_), expected heterozygosity (*H*_E_) and nucleotide diversity (π). As estimates of genetic diversity are highly inter‐correlated (*r* > 0.9, *p* < 0.01), we focused all downstream analyses on nucleotide diversity (π). First, we used Levene's tests to examine whether variances in population genetic diversity differ across species (e.g., Ortego, Gugger, and Sork 2015). Second, we analysed the genetic diversity of populations in relation to (i) geographical peripherality, (ii) elevation and (iii) pond area. Geographic peripherality was estimated as the geodesic distance of each population to the species' distribution centroid. The centroid of species distribution was calculated in ArcMap v. 10.8 on the basis of a minimum convex polygon including all known occurrences of each focal species within the Sierra Nevada mountain range (e.g., Noguerales et al. 2016). We analysed the data using generalised linear models (GLMs) with a Gaussian error distribution and an identity link function, as implemented in the R package ‘lme4’ (Bates et al. 2015). Because the precision of genetic diversity estimates may vary among populations due to differences in sample sizes, we applied a weighted least‐squares (WLS) method, where weight equals the number of genotyped individuals per population (Table S1). To identify the explanatory variables that best accounted for variation in genetic diversity, we used an information theoretic model selection approach based on Akaike's Information Criterion corrected for small sample sizes (AIC_c_; Burnham and Anderson 2002). Models were ranked according to their AIC_c_ values, and those models with ΔAIC_c_ ≤ 2 were considered to have similar empirical support to the best‐fitting model (i.e., the model with the lowest AIC_c_). In such cases, only the model with the highest Akaike weight (*ω*_*i*_) was reported. Model selection was performed using the *dredge* function in the R package ‘MuMIn’ (Bartoń 2025).

### 2.6 Past Demographic History

We reconstructed the demographic history of each population using the program stairway plot v. 2.1, which implements a flexible multi‐epoch demographic model based on the site frequency spectrum (SFS) that does not require whole‐genome sequence data or reference genome information (Liu and Fu 2020). Only populations with seven or more genotyped individuals were considered for these analyses (Table S1). To maximise the number of retained SNPs for the calculation of the SFS, we ran the program *populations* from stacks separately for each specific population; we exported one random SNP per RAD locus and retained loci that were represented in at least 50% of the individuals of the focal population (*R* = 0.5). To remove all missing data for the calculation of the SFS and minimise errors in allele frequency estimates, each population was down‐sampled to ca. 75% of individuals using a custom Python script written by Andréa T. Thomaz and available on GitHub (https://github.com/ichthya/ThomazKnowles2020_scripts; accessed at 26/02/2024) (Thomaz and Knowles 2020). We ran stairway plot considering two generations per year (Pallarés et al. 2024) and performing 200 bootstrap replicates to estimate 95% confidence intervals. We considered the mutation rate per site per generation of 2.8 × 10^−9^ estimated for *Drosophila melanogaster* (Keightley et al. 2014), which is similar to the spontaneous mutation rate estimated for the butterfly *Heliconius melpomene* (2.9 × 10^−9^; Keightley et al. 2015).

### 2.7 Eco‐Evolutionary Dynamics

We evaluated the association between intra‐specific demographic trajectories and community‐level dynamics (Vellend and Geber 2005; Lamy et al. 2017; Govaert et al. 2021). To this end, we first used Pearson's rank correlations in SPSS to test for the relationship between population genetic diversity within each taxon (see Section 2.5) and species richness of local macroinvertebrate communities (α‐diversity). Second, we used Mantel tests in R to assess the correlation between genetic differentiation among populations (see Section 2.4) and community dissimilarity (β‐diversity). The whole macroinvertebrate community was sampled at each pond using the same procedure as described in Section 2.1 for diving beetles (see details in Methods S2). Beta diversity among ponds was computed as Sørensen's dissimilarity, which was also additively decomposed into its spatial turnover (Simpson's dissimilarity) and nestedness components following the framework proposed by Baselga (2010) and implemented in the R package ‘betapart’ (Baselga and Orme 2012).

### 2.8 Morphological Data

The four studied species are aerial dispersers, as they have well‐developed hind wings, but information regarding their flight capacity is currently unavailable. Since dispersal is shaped by a combination of morphological, physiological and ecological factors, obtaining precise estimates of dispersal ability is challenging, so we used an indirect approximation and compared flight morphology of the four species to evaluate their relative dispersal capacity. The area of the membranous wings and elytra was measured for a representative number of specimens of the four species studied (*n* ≥ 30). Since previous research on Dytiscidae has shown sexual dimorphism in different morphological traits (e.g., Bilton et al. 2008, 2016; Liao and Lin 2024), we balanced the number of individuals between sexes: *Agabus nevadensis* (♀ = 24, ♂ = 17), *Hydroporus marginatus* (♀ = 15, ♂ = 15), *Hydroporus sabaudus sierranevadensis* (♀ = 16, ♂ = 14) and *Boreonectes ibericus* (♀ = 15, ♂ = 18). The right wing was removed, spread and mounted on a microscope slide in a 50% dimethyl hydantoin formaldehyde (DMHF) solution. Similarly, the right elytron was removed from each individual. Wings and elytra were photographed under a Motic SMZ‐168 stereomicroscope using a Canon EOS 250D, and wing and elytron area were estimated using ImageJ v.1.54 (Abramoff et al. 2004). We used elytron area as a proxy for body size and the ratio between elytron area and hindwing area as a proxy for wing loading (for a similar approach, see Arribas, Velasco, et al. 2012). Lower wing loadings are related to a higher flight capacity in insects (Rundle et al. 2007). Finally, we performed non‐parametric Kruskal‐Wallis tests in R to compare differences in both traits among the four taxa and used post hoc Bonferroni‐corrected Dunn's tests to examine differences between each pair of taxa. We also performed these comparisons separately for each sex.

## 3 Results

### 3.1 Genomic Datasets

After filtering loci as detailed in Section 2.2, the final data sets retained 6282 SNPs for *A. nevadensis* , 1985 SNPs for *H. marginatus* , 7206 SNPs for *H. sabaudus sierranevadensis* and 3416 SNPs for *B. ibericus*. The average proportion of missing data was 13% for *A. nevadensis* (range = 6%–59%), 14% for *H. marginatus* (range = 7%–32%), 15% for *H. sabaudus sierranevadensis* (range = 6%–58%) and 12% for *B. ibericus* (range = 6%–44%). Other attributes of the genomic datasets obtained for each studied species are presented in Table S2.

### 3.2 Population Genetic Structure and Gene Flow

**FIGURE 1:**
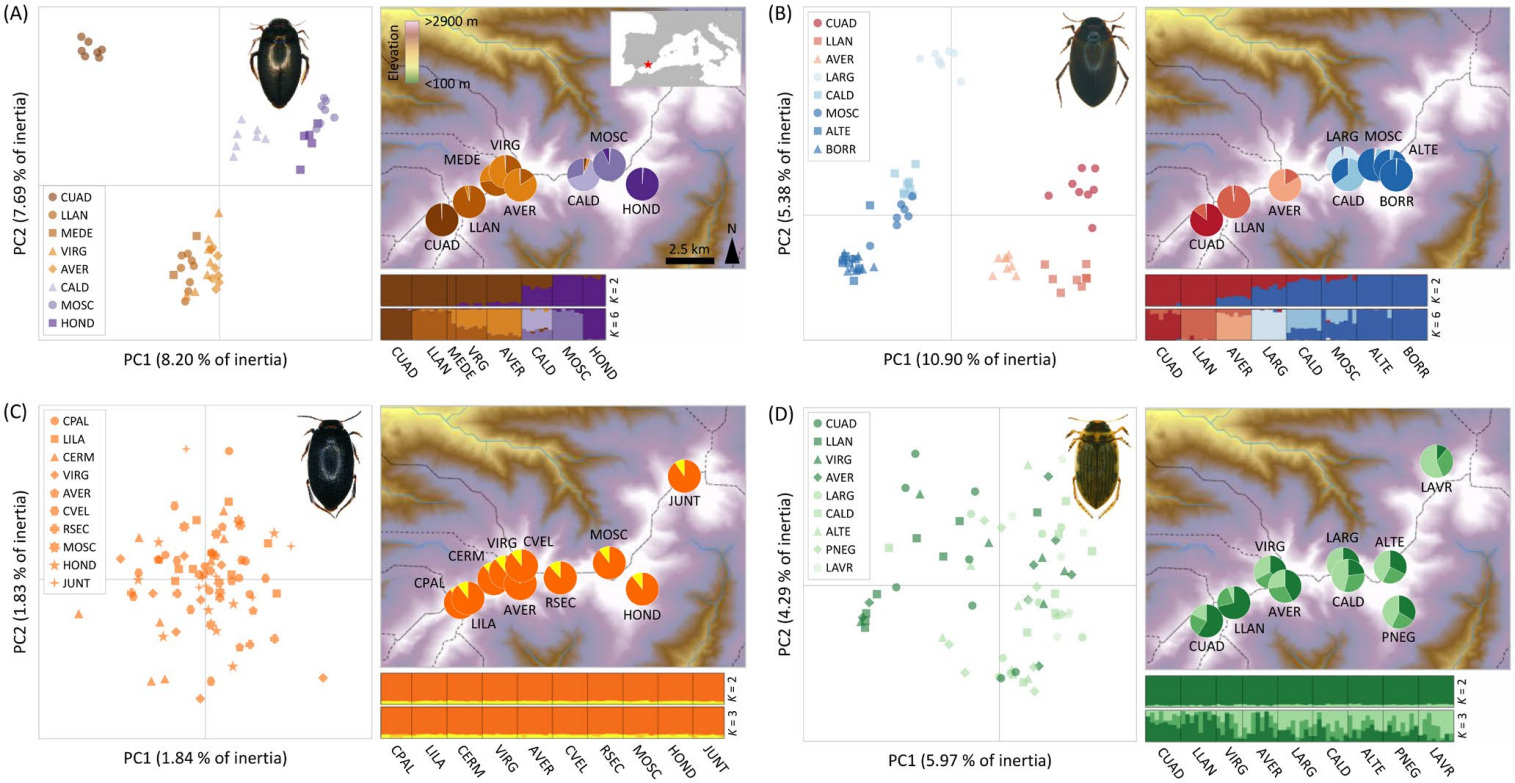
Principal component analyses (PCAs) of genetic variation and results of genetic assignments based on structure for (A) *Agabus nevadensis* (6282 SNPs), (B) *Hydroporus marginatus* (1985 SNPs), (C) *Hydroporus sabaudus sierranevadensis* (7206 SNPs) and (D) *Boreonectes ibericus* (3416 SNPs). Pie charts on maps show the geographic location of populations and their respective genetic assignments. In barplots, each individual is represented by a vertical bar partitioned into *K* coloured segments showing the individual's probability of belonging to the cluster with that colour; thin vertical black lines separate individuals from different populations. Results for other *K*‐values are presented in Figures S2–S5. Dashed lines on maps delineate hydrological basins (https://www.hydrosheds.org/). Pictures for each species by J. A. Carbonell. Population codes as described in Table S1.

structure analyses for *A. nevadensis* identified the most likely number of clusters as *K* = 3 according to the Δ*K* criterion, but LnPr(X|*K*) reached a plateau at *K* = 6 (Figure S1a). For *K* = 2, the two genetic clusters separated populations east and west from Pico del Veleta, with different degrees of genetic admixture in populations located in the contact zone between the two clusters (VIRG, AVER and CALD; Figure 1a). For *K* = 3, the westernmost population CUAD split from the rest of the western populations (Figure S2). Populations of *A. nevadensis* split hierarchically at higher *K*‐values (from *K* = 3 to *K* = 6), presenting different degrees of genetic admixture between nearby populations (Figure 1a and Figure S2). The taxon *H. marginatus* presented a very similar pattern of genetic structure to that reported in *A. nevadensis* . structure analyses for *H. marginatus* identified the most likely number of clusters as *K* = 2 according to the Δ*K* criterion, but LnPr(X|*K*) reached a plateau at *K* = 7 (Figure S1b). For *K* = 2, the two genetic clusters separated populations located east and west from Pico del Veleta (3396 m.a.s.l.), the second‐highest summit in Sierra Nevada mountain range (Figure 1b). Populations located in the contact zone between the two clusters presented a considerable degree of genetic admixture (AVER and LARG; Figure 1b). Populations of *H. marginatus* split hierarchically at higher *K*‐values (from *K* = 3 to *K* = 7), with different degrees of genetic admixture between nearby localities (Figure 1b and Figure S3). structure analyses for *H. sabaudus sierranevadensis* identified the most likely number of clusters as *K* = 2 according to the Δ*K* criterion and LnPr(X|*K*) steadily declined from *K* = 2 to *K* = 10 (Figure S1c). For *K* = 2, all individuals and populations of *H. sabaudus sierranevadensis* presented a very low probability of assignment (*q* < 0.15) to one of the two genetic clusters (i.e., a ‘fictive’ or ‘ghost’ cluster *sensu* Guillot et al. 2005; see also Chen et al. 2007; González‐Serna et al. 2020), which indicates a lack of genetic structure (Figure 1c). Clustering solutions for higher *K*‐values (from *K* = 3 to *K* = 4) did not reveal any genetic structure in *H. sabaudus sierranevadensis* (Figure 1c and Figure S4). Finally, structure analyses for *B. ibericus* showed that Δ*K* peaked at *K* = 2 and *K* = 3 and LnPr(X|*K*) steadily declined from *K* = 3 to *K* = 10 (Figure S1d). For *K* = 2, all individuals and populations of this taxon presented a very low probability of assignment (*q* < 0.1) to one of the two inferred genetic clusters (Figure 1d). However, clustering solutions for *K* = 3 revealed a gradual west‐to‐east gradient of weak genetic differentiation, with considerable genetic admixture among the three inferred genetic clusters (Figure 1d). Clustering solutions for higher *K*‐values (from *K* = 3 to *K* = 4) did not reveal any further genetic structure in *B. ibericus* (Figure 1d and Figure S5). Principal component analyses (PCA) of genetic variation were congruent with the results yielded by Bayesian clustering analyses, showing a genetic clustering of populations and individuals similar to that inferred by structure at the different hierarchical levels for each of the four studied taxa (Figure 1).

Estimates of genetic differentiation (*F*_ST_) between populations of each studied species are presented in Tables S3–S6. For *A. nevadensis*, *F*_ST_ values ranged between 0.044 and 0.319 and all pair‐wise comparisons were significantly different from zero (Table S3). For *H. marginatus* , pair‐wise *F*_ST_ values ranged between 0 and 0.276 and all were significantly different from zero except the comparison involving the nearby populations ALTE and BORR (Table S4). Pair‐wise *F*_ST_ values for *H. sabaudus sierranevadensis* ranged between 0 and 0.019 and were not significantly different from zero in any pair‐wise comparison (Table S5). Finally, pair‐wise *F*_ST_ values for *B. ibericus* ranged between 0 and 0.106 and only some comparisons involving the peripheral populations LLAN and LAVR were significantly different from zero (Table S6).

**FIGURE 2:**
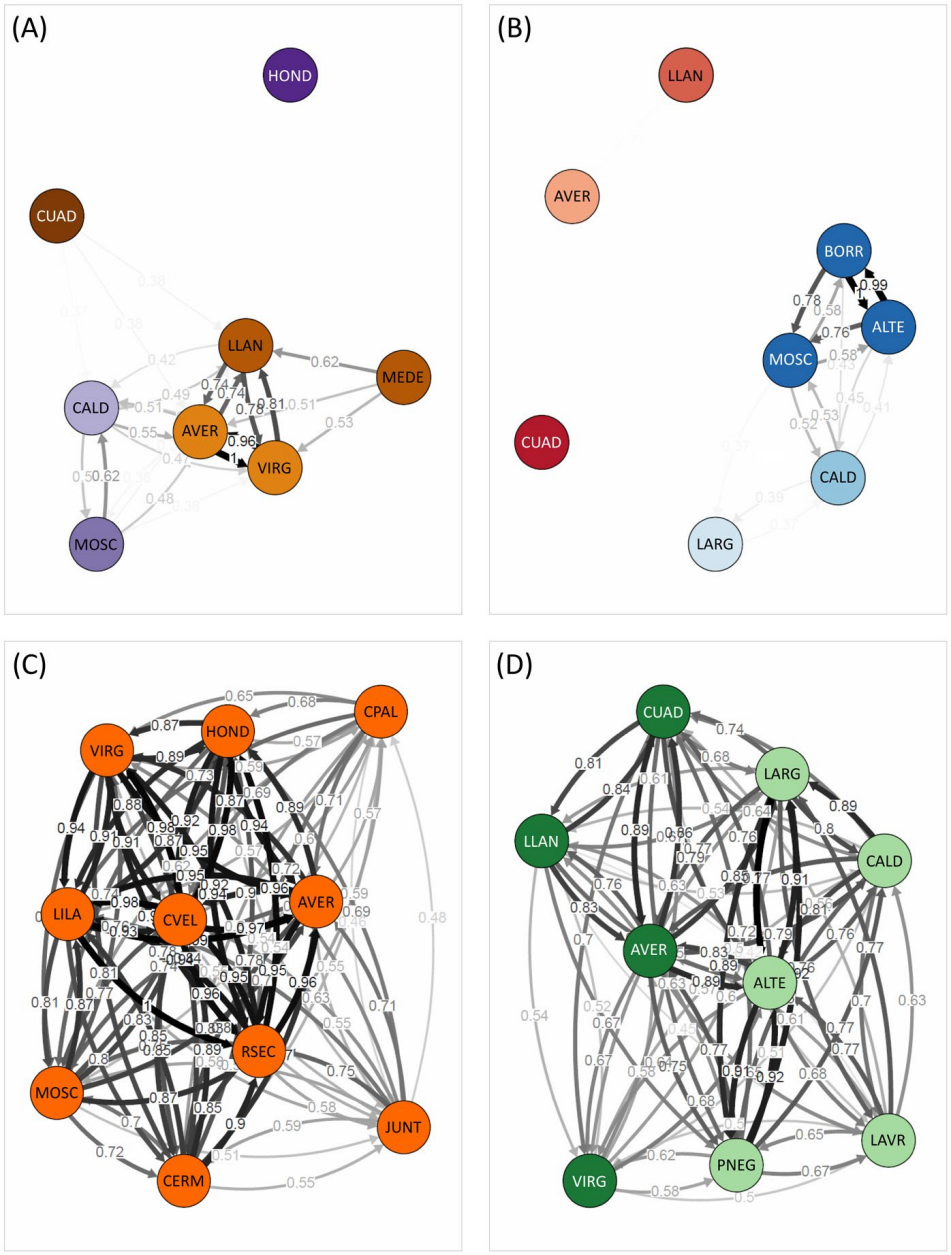
Relative migration networks based on the effective number of migrants (*Nm*) and 1000 bootstraps in *divMigrate* for (A) *Agabus nevadensis* (6282 SNPs), (B) *Hydroporus marginatus* (1985 SNPs), (C) *Hydroporus sabaudus sierranevadensis* (7206 SNPs) and (D) *Boreonectes ibericus* (3416 SNPs). Only significant gene flow between nodes and *Nm* values above the filter threshold of 0.35 are displayed. Node colours correspond to the main genetic cluster at which each population was assigned according to structure analyses for the highest *k*‐value presented in Figure 1. Population codes as described in Table S1.

Relative migration networks estimated with *divMigrate* were congruent with patterns of genetic structure and admixture inferred by structure (Figure 2). Migration networks showed that gene flow in *A. nevadensis* and *H. marginatus* was restricted to certain clusters of nearby populations, with several populations remaining highly isolated in both species (Figure 2a,b). Conversely, *divMigrate* analyses revealed widespread gene flow among populations of *H. sabaudus sierranevadensis* and *B. ibericus* (Figure 2c,d).

### 3.3 Landscape Genetic Analyses

**FIGURE 3:**
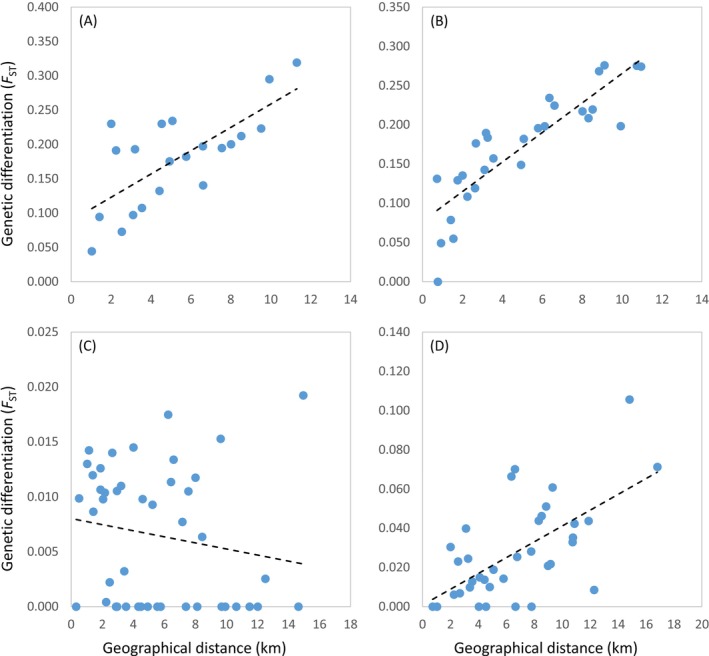
Relationship between genetic differentiation (*F*_ST_) and geographical distances between populations for (A) *Agabus nevadensis* , (B) *Hydroporus marginatus* , (C) *Hydroporus sabaudus sierranevadensis* and (D) *Boreonectes ibericus*. Regression lines are shown.

MMRR showed that genetic differentiation (*F*_ST_) was significantly correlated with both geographical and topographical distances between populations of *A. nevadensis* , *H. marginatus* and *B. ibericus* (Table 1 and Table S7; Figure 3). However, models including geographical distance (Table 1) provided a better fit to the data than those including topographical distance (Table S7). Only in the case of *H. marginatus* , elevation dissimilarity was also retained in the final model (Table 1). For *H. sabaudus sierranevadensis* , no variable was significantly correlated with genetic differentiation (Table 1 and Table S7).

**TABLE 1:** Multiple matrix regressions with randomisation (MMRR) for genetic differentiation (*F*_ST_) between populations in relation to geographical distance and elevation dissimilarity.

Variable	*β*	*t*	*p*
(A) *Agabus nevadensis* (*R* ^2^ = 0.519)			
Explanatory terms			
Constant		1.13	1.000
Geographical distance	0.622	4.53	< 0.001
Rejected terms			
Elevation dissimilarity		−0.28	0.602
(B) *Hydroporus marginatus* (*R* ^2^ = 0.794)			
Explanatory terms			
Constant		0.93	0.976
Geographical distance	0.801	9.79	< 0.001
Elevation dissimilarity	0.174	2.15	0.025
(C) *Hydroporus sabaudus sierranevadensis* (*R* ^2^ = 0.000)			
Rejected terms			
Geographical distance		−1.15	0.862
Elevation dissimilarity		−0.20	0.583
(D) *Boreonectes ibericus* (*R* ^2^ = 0.394)			
Explanatory terms			
Constant		0.03	0.998
Geographical distance	0.680	4.71	0.003
Rejected terms			
Elevation dissimilarity		−0.54	0.667

*Note: R* ^2^, coefficient of determination; *β*, standardised regression coefficient; *t*, *t*‐statistic; *p*, one‐tailed significance level.

### 3.4 Population Genetic Diversity

**FIGURE 4:**
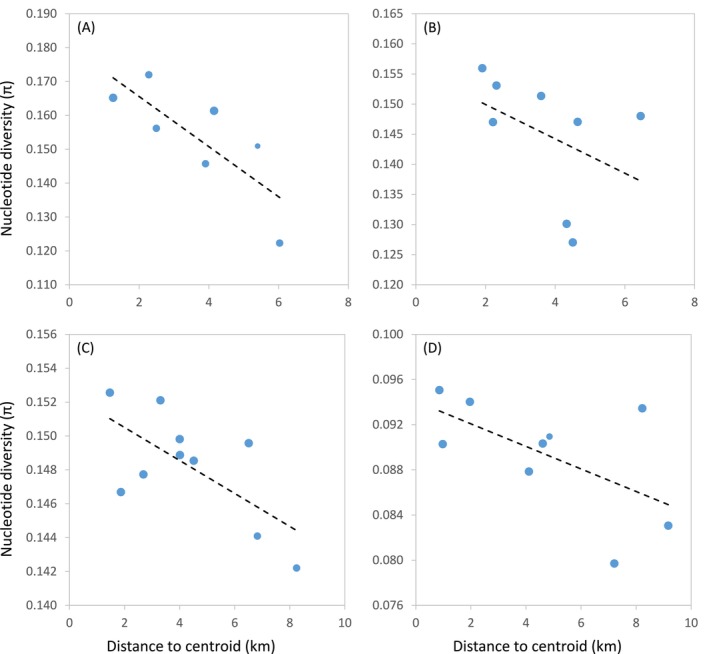
Relationship between the genetic diversity (π) and distance of populations to the centroid of species' distribution within the Sierra Nevada mountain range (i.e., peripherality) for (A) *Agabus nevadensis* , (B) *Hydroporus marginatus* , (C) *Hydroporus sabaudus sierranevadensis* and (D) *Boreonectes ibericus*. Regression lines are shown; dot size is proportional to sample size.

Population genetic statistics (*H*_O_, *H*_E_, *π* and *F*_IS_) calculated for all positions (polymorphic and nonpolymorphic) and only considering variant positions are presented in Table S1. Variances in population genetic diversity differed among species (Levene's test: *F*_3,31_ = 3.89, *p* = 0.018). Pair‐wise Levene's tests showed that variance in population genetic diversity differed significantly between *A. nevadensis* and *H. sabaudus sierranevadensis* (*F*_1,16_ = 7.03, *p* = 0.017) and between *H. marginatus* and *H. sabaudus sierranevadensis* (*F*_1,16_ = 8.22, *p* = 0.011), was marginally significant between *A. nevadensis* and *B. ibericus* (*F*_1,15_ = 4.04, *p* = 0.063) and between *H. marginatus* and *B. ibericus* (*F*_1,15_ = 3.59, *p* = 0.078), and was not significantly different between *H. sabaudus sierranevadensis* and *B. ibericus* (*F*_1,15_ = 1.63, *p* = 0.218). Best‐fitting models for *A. nevadensis* and *H. sabaudus sierranevadensis* indicated that genetic diversity was negatively associated with population peripherality in both species (Table S8 and Table 2; Figure 4). However, for both species, null models (i.e., without explanatory variables) provided a fit similar to that of their respective best‐ranked models (ΔAIC_c_ < 2; Table S10), suggesting that population peripherality only marginally explained variation in genetic diversity. For *B. ibericus* and *H. marginatus* , the best‐fitting model was the null model (Table S8 and Table 2).

**TABLE 2:** Best‐fitting generalised linear model (GLM) for genetic diversity (nucleotide diversity, π).

Variable	Estimate ± SE	*z*	*p*
(A) *Agabus nevadensis* (*R* ^2^ = 0.633)			
Intercept	1.67 × 10^−1^ ± 1.57 × 10^−2^	9.80	< 0.001
Peripherality	−7.55 × 10^−6^ ± 2.57 × 10^−6^	2.24	0.025
(B) *Hydroporus sabaudus sierranevadensis* (*R* ^2^ = 0.416)			
Intercept	1.51 × 10^−1^ ± 2.49 × 10^−3^	56.24	< 0.001
Peripherality	−9.35 × 10^−7^ ± 3.92 × 10^−7^	2.03	0.043

*Note:* For *Hydroporus marginatus* and *Boreonectes ibericus*, the best‐fitting model was the null model (i.e., without explanatory variables; see Table S8). *R* ^2^, coefficient of determination; *β*, standardised regression coefficient; *t*, *z*‐statistic; *p*, significance level.

### 3.5 Past Demographic History

**FIGURE 5:**
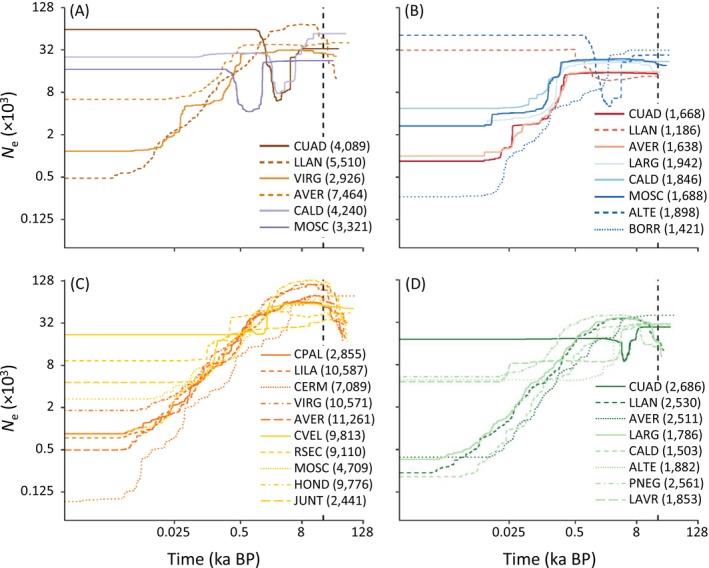
Demographic history of the studied populations of (A) *Agabus nevadensis* , (B) *Hydroporus marginatus* , (C) *Hydroporus sabaudus sierranevadensis* and (D) *Boreonectes ibericus* inferred using stairway plot. Only populations with *n* ≥ 7 genotyped individuals were analysed. Panels show the median of effective population size (*N*_e_) through time, estimated assuming a mutation rate of 2.8 × 10^−9^ and two generations per year (both axes on a logarithmic scale). Vertical dashed line indicates the last glacial maximum (LGM; ~21,000 years ago). The number of polymorphic SNPs used to calculate the site frequency spectrum (SFS) for each population is indicated in parentheses. Colours correspond to the main genetic cluster to which each population was assigned according to structure analyses for the highest *k*‐value presented in Figure 1. Population codes are described in Table S1.

stairway plot analyses revealed that populations of the four studied taxa have undergone contrasting demographic trajectories. Most populations of the genetically weakly structured *H. sabaudus sierranevadensis* and *B. ibericus* experienced parallel changes of *N*_e_ through time, undergoing severe demographic declines starting at the onset of the Holocene (Figure 5). In some cases, these Holocene declines were preceded by demographic expansions during the last glacial period (Figure 5). The only exception was the population CUAD from *B. ibericus*, which experienced a moderate genetic bottleneck ca. 6 ka BP followed by a population recovery. The genetically structured populations of *A. nevadensis* and *H. marginatus* presented more heterogeneous and idiosyncratic demographic dynamics. Several populations of these two taxa experienced demographic declines starting between 0.4 and 8 ka BP (LLAN, VIRG and AVER in *A. nevadensis* and CUAD, AVER, LARG, CALD, MOSC and BORR in *H. marginatus* ; Figure 5). Population LLAN from *H. marginatus* underwent a moderate expansion ca. 1 ka BP followed by demographic stability. Finally, some populations passed through substantial genetic bottlenecks between 3 and 1 ka BP (CUAD, CALD and MOSC in *A. nevadensis* and ALTE in *H. marginatus* ) (Figure 5).

### 3.6 Eco‐Evolutionary Dynamics

Species richness in the studied ponds ranged from 4 to 26 (mean = 13.9; see Table S9). Pair‐wise beta diversity between ponds is presented in Tables S10–S12. Genetic diversity (π) was not correlated with species richness (α‐diversity) in any of the focal taxa (all *p* > 0.492; Table S13). Similarly, genetic differentiation among populations showed no significant correlation with community dissimilarity (β‐diversity) in any taxon, regardless of whether it was estimated using Sørensen's dissimilarity, Simpson's dissimilarity or the nestedness component (all *p* > 0.075; Table S14).

### 3.7 Morphological Data

**FIGURE 6:**
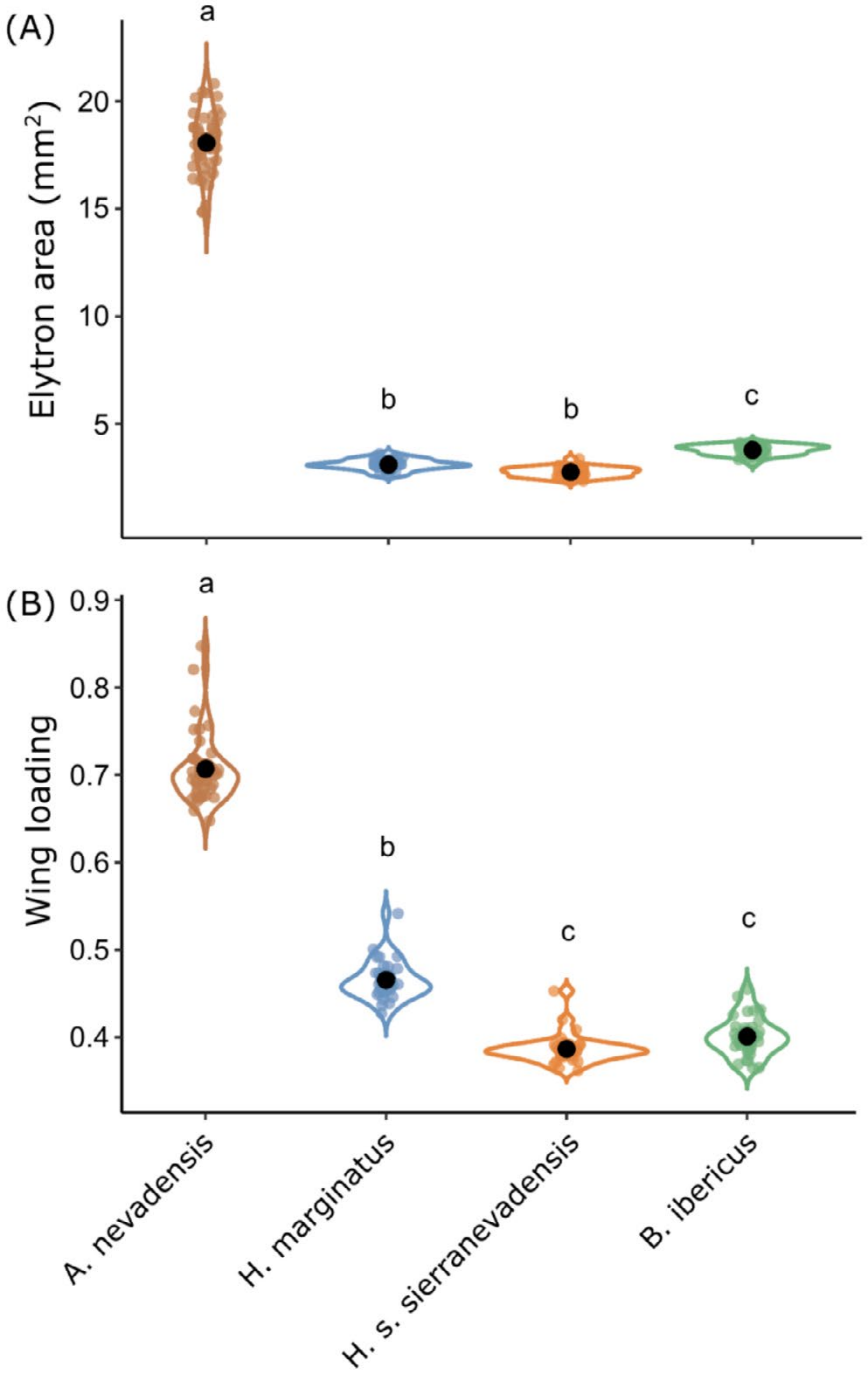
(A) Elytron area and (B) wing loading (elytron area/wing area) for *Agabus nevadensis* (*n* = 41), *Hydroporus marginatus* (*n* = 30), *Hydroporus sabaudus sierranevadensis* (*n* = 30) and *Boreonectes ibericus* (*n* = 33). Violin plots show estimated values for each trait (small coloured dots) and mean and confidence intervals (black dots and vertical bars, respectively). Different lowercase letters above the plots indicate statistically significant differences among taxa based on post hoc Bonferroni‐corrected Dunn's tests (*p* < 0.05).

Kruskal–Wallis tests showed significant differences among taxa in both elytron area (*χ* ^2^ = 118.67, df = 3, *p* < 0.001; Figure 6a) and wing loading (*χ* ^2^ = 114.39, df = 3, *p* < 0.001; Figure 6b). Post hoc tests for elytron area indicated that only the pair‐wise comparison between *H. marginatus* and *H. sabaudus sierranevadensis* (*p* = 0.074) was not significant (Figure 6a). *Agabus nevadensis* had a much larger elytra than the other species, followed by *B. ibericus*, *H. marginatus* and *H. sabaudus sierranevadensis* (Figure 6a). In the post hoc tests for wing loading, only the comparison between *H. sabaudus sierranevadensis* and *B. ibericus* was not statistically significant (*p* = 0.427; Figure 6b). As with elytron area, *A. nevadensis* had a much higher wing loading than the other species, followed by *H. marginatus* , *B. ibericus* and *H. sabaudus sierranevadensis* (Figure 6b). Significant differences between males and females were found only in *A. nevadensis* for both elytron area (*W* = 39, *p* < 0.001) and wing loading (*W* = 113, *p* < 0.015) and in *H. marginatus* for elytron area (*W* = 173, *p* = 0.011), as determined by Wilcoxon tests (Figure S6). When analyses were performed separately for each sex, significant differences among taxa remained in both elytron area (♀: *χ* ^2^ = 61.59, df = 3, *p* < 0.001; ♂: *χ* ^2^ = 56.41, df = 3, *p* < 0.001; Figure S6a) and wing loading (♀: *χ* ^2^ = 59.77, df = 3, *p* < 0.001; ♂: *χ* ^2^ = 53.96, df = 3, *p* < 0.001; Figure S6b).

## 4 Discussion

Our genomic analyses revealed contrasting population connectivity and demographic trajectories among four diving beetles co‐distributed in an alpine lake network from the Sierra Nevada mountain range, southeastern Iberia. While some species showed a marked genetic fragmentation even at small spatial scales (< 4 km), others presented either a complete lack or very subtle genetic structure, with widespread gene flow across the landscape (Figures 1 and 2). Remarkably, such differences were not associated with the species' distributional ranges, as deep and subtle genetic structuring was observed in both local endemic and widely distributed taxa. Morphometric analyses suggest that such contrasting genetic patterns can be explained by differences in dispersal capacity among taxa, with those exhibiting lower wing loadings displaying higher levels of population genetic connectivity.

### 4.1 Drivers of Genetic Structure

Although the four studied taxa share similar ecological requirements, predominantly occupy lentic habitats and disperse via flight, our results reveal substantial heterogeneity in their demographic responses to the naturally fragmented distribution of high‐mountain lakes. Interspecific differences in genetic structure appear to reflect variation in dispersal ability, with species presenting higher wing loadings ( *A. nevadensis* and *H. marginatus* ) showing stronger genetic structuring compared to the subtle or absent differentiation in taxa with lower wing loadings ( *H. sabaudus sierranevadensis* and *B. ibericus*) (Figure 6). Despite the topographic complexity of the landscape, our spatially explicit analyses showed that genetic differentiation among populations of *A. nevadensis* , *H. marginatus* , and, to a lesser extent, *B. ibericus* is primarily driven by the geographical distances separating them (Figure 3), a typical pattern of isolation‐by‐distance (IBD) arising from a balance between gene flow and genetic drift (Wright 1943; Hutchison and Templeton 1999). In contrast, widespread gene flow appears to be the dominant force shaping the genetic homogeneity observed in *B. ibericus* (see also Phillipsen et al. 2015). The minimal influence of the rest of the landscape variables on spatial patterns of genetic differentiation is likely due to the aerial dispersal strategy of the studied taxa, resulting in genetic structure—or lack thereof—being primarily determined by dispersal rates and the species' capacity to bridge straight‐line geographic distances between lakes (Figure 2). Apart from geographic distance, only elevation dissimilarity explained genetic differentiation in *H. marginatus* , which might reflect differences in phenology (i.e., isolation‐by‐time; Hendry and Day 2005) and/or local adaptations and selection against immigrants in populations experiencing contrasting environmental conditions at different altitudinal ranges (i.e., isolation‐by‐environment; Sexton et al. 2014; Wang and Bradburd 2014).

### 4.2 Implications of Contrasting Genetic Structure

Lentic habitats, such as lakes and ponds, are typically ephemeral on evolutionary timescales, often leading to increased extinction risk and selecting for more dispersive phenotypes (Marten et al. 2006; e.g., Hjalmarsson et al. 2015; Abellán et al. 2009). However, our results revealed strikingly different patterns of genetic structure and connectivity among the four co‐distributed taxa, challenging the hypothesis that lentic‐habitat specialists evolve high dispersal capacities (Marten et al. 2006; Ribera 2008). Several factors may explain this apparent discrepancy between our findings and those reported in previous studies (Marten et al. 2006; Ribera 2008; Abellán et al. 2009; Hjalmarsson et al. 2015; see, however, Short and Caterino 2009; Phillipsen et al. 2015). First, most previous studies have relied on gene fragments (e.g., mtDNA; Abellán et al. 2009; Hjalmarsson et al. 2015) to infer range dynamics and dispersal rates at deeper evolutionary timescales (i.e., phylogeographic) than those addressed by our contemporary landscape‐level genomic analyses. Second, the lentic‐lotic dichotomy regarding dispersal and genetic structure frames within the broader ‘habitat constraint’ hypothesis, which predicts that the persistence of taxa inhabiting dynamic and unstable habitats requires frequent inter‐patch migration (Southwood 1962; see Meramveliotakis et al. 2024). Although water levels in Sierra Nevada's alpine lakes fluctuate seasonally and yearly, most are lentic systems that have remained permanent and relatively stable since their formation after the last glacial retreat (Castillo Martín 2009). This contemporary stability of a geologically ephemeral habitat might have facilitated the coexistence of contrasting dispersal strategies in either local endemic or widely distributed taxa.

For instance, *A. nevadensis* is a neo‐endemic taxon that diverged < 15 ka BP from an Iberian lineage of the habitat generalist *A. bipustulatus* (Pallarés et al. 2024), coinciding with the formation of Sierra Nevada's alpine lakes at the end of the last glacial period (Castillo Martín 2009). At the evolutionary timescale of *A. nevadensis* , the network of alpine lakes has represented a geographically restricted and relatively stable habitat. In line with the ‘habitat constraint’ hypothesis (Southwood 1962), such habitat stability may have favoured the evolution of reduced dispersal in this recently originated species (Waters et al. 2020). Conversely, older lineages may have colonised alpine lakes from typically unstable lentic habitats after the last glacial period, potentially decoupling their dispersive phenotypes from the stability of the habitats they currently occupy. This is exemplified in *B. ibericus* and *H. sabaudus sierranevadensis* , which belong to clades of lentic‐habitat specialists that originated > 10 Ma BP, a timescale largely predating the formation of the network of alpine lakes in Sierra Nevada (Villastrigo et al. 2021).

Interspecific differences in dispersal ability and genetic structure can also shed some light on the proximate processes underlying the origin and persistence of the two Sierra Nevada endemics, *A. nevadensis* and *H. sabaudus sierranevadensis* . The distribution of *A. nevadensis* is entirely embedded within the broad distribution range of its sister species *A. bipustulatus* and hybridisation between the two taxa is pervasive in some areas of northeastern Sierra Nevada, where some populations form hybrid swarms (Pallarés et al. 2024). Under this scenario, the very limited dispersal capacity of *A. nevadensis* might have been instrumental not only in promoting isolation at the onset of speciation (i.e., lineage formation) but also in preventing speciation reversal through hybridisation with *A. bipustulatus* (i.e., lineage persistence) (Dynesius and Jansson 2014). In contrast, available phylogenetic evidence indicates that the closest relative of the taxon *H. sabaudus sierranevadensis* is the Balkan–Anatolian–Caucasian *H. thracicus* Guéorguiev, 1966 (Villastrigo et al. 2021), distributed > 2000 km away from Sierra Nevada (Shaverdo 2004). Dating analyses indicate that these two taxa probably diverged ca. 1.2 Ma BP, suggesting that *H. sabaudus sierranevadensis* might be a relict endemic species that once had a broader distribution (Villastrigo et al. 2021). The taxon *H. sabaudus sierranevadensis* presents an allopatric distribution unreachable by any close relative with which it could potentially hybridise (Shaverdo 2004). For this reason, the high dispersal capacity of this taxon cannot compromise its genetic integrity through hybridisation with closely related taxa, even if reproductive isolation is incomplete (Shaverdo 2004). Altogether, this exemplifies how the interplay among geological events (i.e., isolation driven by Pleistocene glacial cycles), biogeographical history (i.e., range dynamics) and organismal traits (i.e., dispersal capacity) results in contrasting pathways through which micro‐endemic species originated and persist in alpine ecosystems (Dynesius and Jansson 2014).

### 4.3 Demographic Trajectories of Populations

Consistent with the ‘centre‐periphery’ hypothesis (Sexton et al. 2009), populations located at the range peripheries within Sierra Nevada tended to exhibit lower genetic diversity than central populations (Figure 4). This pattern, previously reported for other alpine organisms endemic to Sierra Nevada and adjacent mountain ranges (Tonzo and Ortego 2021), likely reflects the continuous contraction of alpine habitats since the end of the last glacial period and reduced demographic performance of cold‐adapted organisms toward their ecological limits (Sexton et al. 2009; Lira‐Noriega and Manthey 2014; Pironon et al. 2017). Remarkably, we found no effect of lake size on local levels of genetic diversity. This could be explained by the large effective population sizes that can be sustained by water beetles even in small pools in Sierra Nevada (Millán et al. 2013; Abellán et al. 2022), which could decouple genetic drift from habitat patch size. Despite this general pattern, taxa showing stronger genetic structure also displayed higher among‐population variance in genetic diversity and presented more heterogeneous demographic trajectories, likely due to disrupted gene flow and long‐term isolation of their populations (e.g., Ortego, Gugger, and Sork 2015). It must be noted, however, that the limited number of populations analysed (*n* = 7–10; Table S1), inherently constrained by the small number of alpine lakes where each taxon occurs within the restricted geographic extent of the Sierra Nevada massif, may have reduced the statistical power of our analyses of genetic diversity.

Demographic reconstructions in stairway plots revealed a general decline in effective population size (*N*_e_) from the last glacial maximum (LGM) to present (Figure 6), as expected for cold‐adapted species that likely sustained larger and more connected populations during glacial periods and became confined to mountain tops during interglacials (Tonzo and Ortego 2021; Ortego and Knowles 2022). However, some populations of *A. nevadensis* , *H. marginatus* and *B. ibericus* experienced bottlenecks that, according to paleoclimatic reconstructions for Sierra Nevada alpine lakes, aligned with the warmer climate and lower lake levels that characterised the Holocene Thermal Maximum (HTM; ca. 9–7.2 ka BP) and the Medieval Climate Anomaly (MCA; ca. 1 ka BP) (Jiménez‐Moreno et al. 2023; López‐Blanco et al. 2024). This highlights the higher sensitivity of species with more limited dispersal capacity to environmental fluctuations, which can result in isolated populations experiencing marked demographic declines when lake levels drop or ecological conditions worsen during dry and warmer periods.

### 4.4 Eco‐Evolutionary Community Dynamics

The parallelism between the main processes that operate at evolutionary and community ecology scales has been proposed to result in a dynamical interplay between intra‐specific demographic trajectories and community‐level dynamics in groups of ecologically similar species (Vellend and Geber 2005; Lamy et al. 2017; see figure 1 in Govaert et al. 2021). The parallelism between such processes has been hypothesised to be reflected in positive correlations between neutral genetic diversity in populations of focal species and species diversity in local communities (α‐diversity) and between genetic differentiation among populations within species and community dissimilarity (β‐diversity). Despite these correlations being expected to be stronger in island‐like habitats such as alpine lakes (Vellend and Geber 2005; Vellend et al. 2014; Lamy et al. 2017), the composition of macroinvertebrate communities (α‐diversity and β‐diversity) was not associated with intra‐specific levels of genetic diversity or connectivity in either studied species. This decoupling may stem from multiple factors (Lamy et al. 2017), including limited statistical power due to low variance in genetic diversity and differentiation in taxa with widespread gene flow, or differences in community successional stages driven by the interplay among environmental gradients (Abellán et al. 2022), the recent origin of glacial lakes (Díaz‐Hernández and Herrera‐Martínez 2021) and heterogeneity in fluctuating hydrological regimes since lake formation (Castillo Martín 2009; Jiménez‐Moreno et al. 2023; López‐Blanco et al. 2024).

## 5 Conclusions

This study shows that aquatic beetle species co‐occurring in an alpine lake network exhibit contrasting patterns of genetic structure and demographic dynamics, reflecting interspecific differences in dispersal capacity rather than shared environmental constraints. Our findings emphasise that even within ecologically similar communities, species may follow distinct evolutionary and demographic trajectories shaped by their dispersal abilities and specific responses to landscape structure. The different evolutionary and biogeographical histories of the focal species, together with the recent formation of alpine lakes, might have contributed to the coexistence of contrasting dispersive strategies. The fact that species with similar habitat requirements and regional distributions respond idiosyncratically to habitat fragmentation hinders the generalisation of conservation strategies, but the obtained results indicate that these should focus on the long‐term population monitoring of taxa with limited dispersal capacities and, thus, more prone to experience local extinctions (e.g., lake dry out) unlikely to be reverted through natural re‐colonisation from standing populations (Lamouille‐Hébert et al. 2024; Pallarés et al. 2020). This takes special relevance under ongoing climate change, which is particularly amplified at higher elevations in Sierra Nevada and will likely compromise the persistence of alpine lakes and their associated communities through the alteration of hydrological dynamics (Jiménez‐Moreno et al. 2023). Collectively, our study highlights the importance of multi‐taxon approaches to understand community‐level demographic dynamics in biodiversity hotspots such as Mediterranean alpine ecosystems highly sensitive to climate warming. Future studies encompassing larger networks of alpine lakes, expanding analyses to other assemblages of co‐distributed organisms and incorporating more precise demographic reconstructions based on whole‐genome data (e.g., Santiago et al. 2020) may further improve our understanding of the demographic dynamics of alpine lake communities.

## Author Contributions

J.O. and P.A. conceived and designed the study. P.A., S.P. and J.A.C. collected the samples. E.F.‐F. and J.O. prepared the genomic libraries. D.C.‐F. and P.A. performed morphometric measurements and analyses. J.O. analysed the genomic data. J.O. wrote the manuscript, with inputs from P.A. All authors read, revised and approved the manuscript.

## Conflicts of Interest

The authors declare no conflicts of interest.

## Supporting information


**Figure S1:** Log probability of the data and the magnitude of Δ*K* for structure analyses.
**Figure S2:** Genetic assignments based on structure analyses for *Agabus nevadensis*.
**Figure S3:** Genetic assignments based on structure analyses for *Hydroporus marginatus*.
**Figure S4:** Genetic assignments based on structure analyses for *Hydroporus sabaudus sierranevadensis*.
**Figure S5:** Genetic assignments based on structure analyses for *Boreonectes ibericus*.
**Figure S6:** Elytron area and wing loading for females and males of each studied species.
**Table S1:** Sampling sites and genetic diversity statistics for populations of each studied species.
**Table S2:** Attributes of genomic datasets obtained for each studied species.
**Table S3:** Genetic differentiation (*F*_ST_) between populations of *Agabus nevadensis* .
**Table S4:** Genetic differentiation (*F*_ST_) between populations of *Hydroporus marginatus*.
**Table S5:** Genetic differentiation (*F*_ST_) between populations of *Hydroporus sabaudus sierranevadensis*.
**Table S6:** Genetic differentiation (*F*_ST_) between populations of *Boreonectes ibericus*.
**Table S7:** Multiple matrix regressions with randomisation (MMRR) for genetic differentiation (*F*_ST_).
**Table S8:** Model selection to assess the relationship between genetic diversity and geographical peripherality of populations, elevation and pond area.
**Table S9:** Taxa collected in the ponds studied in Sierra Nevada Massif.
**Table S10:** Pair‐wise beta diversity among ponds computed as Sørensen's dissimilarity.
**Table S11:** Pair‐wise beta diversity among ponds computed as Simpson's dissimilarity.
**Table S12:** Pair‐wise beta diversity among ponds computed as nestedness.
**Table S13:** Correlations between population genetic diversity (π) and species richness (α diversity) of the local communities.
**Table S14:** Correlations between genetic differentiation (*F*_ST_) among populations and community dissimilarity (β‐diversity).
**Methods S1:** Genomic data filtering and assembling.
**Methods S2:** Macroinvertebrate community data.

## Data Availability

Raw Illumina reads have been deposited at the NCBI Sequence Read Archive (SRA) under BioProject PRJNA1111346. Datasets (morphological data) and input files for all analyses (structure, PCAs, *divMigrate*, arlequin, MMRR, and stairway plot) are available for download on Figshare (https://doi.org/10.6084/m9.figshare.29490149).
