# Supplementary material for: Dispersal Capacity Rather Than Shared Environmental Constraints Determines Taxon‐Specific Demographic Dynamics in an Alpine Lake Network
Source: Mol Ecol. 2025 Nov 13;34(24):e70173. doi: 10.1111/mec.70173 (PMC12717978; doi:10.1111/mec.70173)
Supplement: Supplementary file 1 — Figure S1: Log probability of the data and the magnitude of ΔK for structure analyses. Figure S2: Genetic assignments based on structure analyses for Agabus nevadensis. Figure S3: Genetic assignments based on structure analyses for Hydroporus marginatus. Figure S4: Genetic assignments based on structure analyses for Hydroporus sabaudus sierranevadensis. Figure S5: Genetic assignments based on structure analyses for Boreonectes ibericus. Figure S6: Elytron area and wing loading for females and males of each studied species. Table S1: Sampling sites and genetic diversity statistics for populations of each studied species. Table S2: Attributes of genomic datasets obtained for each studied species. Table S3: Genetic differentiation (F ST) between populations of Agabus nevadensis . Table S4: Genetic differentiation (F ST) between populations of Hydroporus marginatus. Table S5: Genetic differentiation (F ST) between populations of Hydroporus sabaudus sierranevadensis. Table S6: Genetic differentiation (F ST) between populations of Boreonectes ibericus. Table S7: Multiple matrix regressions with randomisation (MMRR) for genetic differentiation (F ST). Table S8: Model selection to assess the relationship between genetic diversity and geographical peripherality of populations, elevation and pond area. Table S9: Taxa collected in the ponds studied in Sierra Nevada Massif. Table S10: Pair‐wise beta diversity among ponds computed as Sørensen's dissimilarity. Table S11: Pair‐wise beta diversity among ponds computed as Simpson's dissimilarity. Table S12: Pair‐wise beta diversity among ponds computed as nestedness. Table S13: Correlations between population genetic diversity (π) and species richness (α diversity) of the local communities. Table S14: Correlations between genetic differentiation (F ST) among populations and community dissimilarity (β‐diversity). Methods S1: Genomic data filtering and assembling. Methods S2: Macroinvertebrate community data. [file MEC-34-e70173-s001.docx]

Supporting Information for

**Differences in dispersal capacity rather than shared environmental constraints determine taxon-specific demographic dynamics in an alpine lake network**

Joaquín Ortego, Eduardo Franco-Fuentes, Susana Pallarés, José A. Carbonell, Daniel Caballero-Fernández, and Pedro Abellán

Journal: *Molecular Ecology*

**Contents:**

**Supplementary methods**

**Methods S1** Genomic data filtering and assembling

**METHODS S2** Macroinvertebrate community data

**Supplementary tables**

**Table S1** Sampling sites and genetic diversity statistics for populations of each studied species

**TABLE S2** Attributes of genomic datasets obtained for each studied species

**Table S3** Genetic differentiation (*F*_ST_) between populations of *Agabus nevadensis*

**Table S4** Genetic differentiation (*F*_ST_) between populations of *Hydroporus marginatus*

**Table S5** Genetic differentiation (*F*_ST_) between populations of *Hydroporus sabaudus sierranevadensis*

**Table S6** Genetic differentiation (*F*_ST_) between populations of *Boreonectes ibericus*

**Table S7** Multiple matrix regressions with randomization (MMRR) for genetic differentiation (*F*_ST_)

**TABLE S8** Model selection to assess the relationship between genetic diversity and geographical peripherality of populations, elevation, and pond area.

**TABLE S9** Taxa collected in the ponds studied in Sierra Nevada Massif.

**TABLE S10** Pairwise beta diversity among ponds computed as Sørensen’s dissimilarity.

**TABLE S11** Pairwise beta diversity among ponds computed as Simpson´s dissimilarity.

**TABLE S12** Pairwise beta diversity among ponds computed as nestedness.

**TABLE S13** Correlations between population genetic diversity (π) and species richness (α diversity) of the local communities

**TABLE S14** Correlations between genetic differentiation (*F*_ST_) among populations and community dissimilarity (β-diversity)

**Supplementary figures**

**figure S1** Log probability of the data and the magnitude of Δ*K* for structure analyses

**Figure S2** Genetic assignments based on structure analyses for *Agabus nevadensis*

**Figure S3** Genetic assignments based on structure analyses for *Hydroporus marginatus*

**Figure S4** Genetic assignments based on structure analyses for *Hydroporus sabaudus sierranevadensis*

**Figure S5** Genetic assignments based on structure analyses for *Boreonectes ibericus*

**Figure S6** Elytron area and wing loading for females and males of each studied species

**References**

**Supplementary methods**

**METHODS S1** Genomic data filtering and assembling

We used the different programs distributed as part of the stacks v. 2.66 pipeline (*process_radtags*, *ustacks*, *cstacks*, *sstacks*, and *populations*) to assemble our sequences into *de novo* loci and call genotypes (Rochette et al., 2019). We demultiplexed and filtered reads for overall quality using the program *process_radtags*, retaining those reads with a Phred score > 10 (using a sliding window of 15%), no adaptor contamination, and with an unambiguous barcode and restriction cut site. We screened raw reads for quality with fastqc v. 0.11.5 (<http://www.bioinformatics.babraham.ac.uk/projects/fastqc/>) and trimmed all sequences to 180-bp using trimmomatic v. 0.36 (Bolger et al., 2014) in order to remove low-quality reads near the 3´ ends. We assembled reads *de novo* into putative loci with the program *ustacks*. We set the minimum stack depth (*m*) to three and allowed a maximum distance of two nucleotide mismatches (*M*) to group reads into a “stack”. We used the “removal” (*r*) and “deleveraging” (*d*) algorithms to eliminate highly repetitive stacks and resolve over-merged loci, respectively. We identified single nucleotide polymorphisms (SNPs) at each locus and called genotypes using a multinomial-based likelihood model that accounts for sequencing errors, with the upper bound of the error rate (*ε*) set to 0.2. A conservative upper bound was selected for the error parameter, as these models have been developed primarily for higher coverage data. Using a conservative bound was preferred over the unbounded model, which has been shown to underestimate heterozygotes (Catchen et al., 2013; e.g., Papadopoulou & Knowles, 2015). We built a catalogue of loci using the *cstacks* program, with loci recognized as homologous across individuals if the number of nucleotide mismatches between consensus sequences (*n*) was ≤1. We used the programs *tsv2bam* and *gstacks* with default parameters to transpose the data to be oriented by locus and call variable sites across individuals. Finally, we calculated genetic diversity statistics and exported output files in different formats for subsequent analyses using the program *populations*. Unless otherwise indicated, for all downstream analyses we exported only one random SNP per RAD locus (option *write-random-snp*) and retained loci that were represented in at least 75% of individuals (*R* = 0.75). We used the option *relatedness2* in vcftools to calculate the relatedness among all pairs of genotyped individuals and to exclude the possibility that we had sampled close relatives within each locality (Danecek et al., 2011; Manichaikul et al., 2010). All pairs of genotyped individuals from the four taxa had negative relatedness values, which excludes the possibility of a close relationship (Manichaikul et al., 2010).

**METHODS S2** Macroinvertebrate community data

Macroinvertebrate community data were collected during extensive field surveys conducted in July and August of 2020 and 2021, yielding one macroinvertebrate sample from each of the 19 lakes studied (see Table S1). Sampling was carried out using lake nets (pentagonal, 30 cm deep, 0.5 mm mesh), ensuring that all available microhabitat types were proportionally represented. Each kick sample was transferred to a tray and examined on site, with additional samples taken until no new taxa (morphotypes) were detected. Specimens were preserved in 96% ethanol and subsequently transported to the laboratory for detailed species identification.

**Supplementary tables**

**TABLE S1** Geographical location, number of genotyped individuals (*n*), and genetic summary statistics (*H*_O_, *H*_E_, π, and *F*_IS_) for the populations of each studied species of diving beetle in Sierra Nevada mountain range.

|  |  |  |  |  |  | All positions | | | |  | Variant positions | | | |
| --- | --- | --- | --- | --- | --- | --- | --- | --- | --- | --- | --- | --- | --- | --- |
| Locality | Code | Latitude | Longitude | Elevation | *n* | *H*_O_ | *H*_E_ | π | *F*_IS_ |  | *H*_O_ | *H*_E_ | π | *F*_IS_ |
| (A) *Agabus nevadensis* |  |  |  |  |  |  |  |  |  |  |  |  |  |  |
| Laguna Cuadrada | CUAD | 37.02707 | -3.41860 | 2913 | 7 | 0.0005 | 0.0005 | 0.0006 | 0.0002 |  | 0.0998 | 0.1121 | 0.1223 | 0.0526 |
| Laguna de Lanjarón | LLAN | 37.03772 | -3.40045 | 2981 | 8 | 0.0006 | 0.0007 | 0.0007 | 0.0004 |  | 0.1216 | 0.1496 | 0.1614 | 0.0986 |
| Lagunillo Medio de la Ermita | MEDE | 37.04990 | -3.38499 | 2869 | 2 | – | – | – | – |  | – | – | – | – |
| Lagunillas de la Virgen | VIRG | 37.05265 | -3.37886 | 2946 | 7 | 0.0006 | 0.0007 | 0.0008 | 0.0004 |  | 0.1317 | 0.1559 | 0.1720 | 0.0941 |
| Laguna de Aguas Verdes | AVER | 37.04867 | -3.36838 | 3052 | 8 | 0.0006 | 0.0007 | 0.0008 | 0.0004 |  | 0.1287 | 0.1536 | 0.1652 | 0.0908 |
| Laguna de la Caldera | CALD | 37.05457 | -3.32921 | 3015 | 7 | 0.0005 | 0.0006 | 0.0007 | 0.0004 |  | 0.1202 | 0.1415 | 0.1562 | 0.0825 |
| Laguna de la Mosca | MOSC | 37.05976 | -3.31472 | 2893 | 7 | 0.0005 | 0.0006 | 0.0007 | 0.0004 |  | 0.1136 | 0.1333 | 0.1457 | 0.0764 |
| Laguna Hondera | HOND | 37.04794 | -3.29423 | 2892 | 5 | 0.0005 | 0.0006 | 0.0007 | 0.0003 |  | 0.1175 | 0.1325 | 0.1509 | 0.0689 |
| (B) *Hydroporus marginatus* |  |  |  |  |  |  |  |  |  |  |  |  |  |  |
| Laguna Cuadrada | CUAD | 37.02707 | -3.41860 | 2913 | 8 | 0.0005 | 0.0006 | 0.0007 | 0.0004 |  | 0.1107 | 0.1367 | 0.1480 | 0.0915 |
| Laguna de Lanjarón | LLAN | 37.03772 | -3.40045 | 2981 | 8 | 0.0005 | 0.0006 | 0.0007 | 0.0004 |  | 0.1065 | 0.1352 | 0.1471 | 0.0945 |
| Laguna de Aguas Verdes | AVER | 37.04867 | -3.36838 | 3052 | 8 | 0.0005 | 0.0007 | 0.0007 | 0.0005 |  | 0.1094 | 0.1440 | 0.1560 | 0.1140 |
| Laguna Larga | LARG | 37.05956 | -3.33441 | 2781 | 8 | 0.0005 | 0.0007 | 0.0007 | 0.0004 |  | 0.1187 | 0.1420 | 0.1531 | 0.0826 |
| Laguna de la Caldera | CALD | 37.05457 | -3.32921 | 3015 | 8 | 0.0005 | 0.0006 | 0.0007 | 0.0004 |  | 0.1099 | 0.1357 | 0.1470 | 0.0938 |
| Laguna de la Mosca | MOSC | 37.05976 | -3.31472 | 2893 | 8 | 0.0005 | 0.0006 | 0.0007 | 0.0005 |  | 0.1099 | 0.1397 | 0.1514 | 0.1032 |
| Laguna Altera | ALTE | 37.05850 | -3.30446 | 3065 | 8 | 0.0005 | 0.0006 | 0.0006 | 0.0003 |  | 0.1020 | 0.1195 | 0.1301 | 0.0688 |
| Laguna del Borreguil | BORR | 37.05277 | -3.29977 | 2980 | 8 | 0.0005 | 0.0005 | 0.0006 | 0.0003 |  | 0.0996 | 0.1169 | 0.1271 | 0.0690 |
| (C) *Hydroporus sabaudus sierranevadensis* | | |  |  |  |  |  |  |  |  |  |  |  |  |
| Lagunillo de Charca Pala | CPAL | 37.03596 | -3.40477 | 2944 | 7 | 0.0005 | 0.0007 | 0.0007 | 0.0005 |  | 0.1031 | 0.1282 | 0.1441 | 0.0970 |
| Lagunillo de Lanjarón | LILA | 37.03736 | -3.40164 | 2979 | 8 | 0.0006 | 0.0007 | 0.0008 | 0.0005 |  | 0.1119 | 0.1391 | 0.1496 | 0.1010 |
| Charca de la Ermita | CERM | 37.05093 | -3.38411 | 2867 | 8 | 0.0005 | 0.0007 | 0.0008 | 0.0006 |  | 0.1038 | 0.1370 | 0.1485 | 0.1166 |
| Lagunillas de la Virgen | VIRG | 37.05265 | -3.37886 | 2946 | 8 | 0.0005 | 0.0007 | 0.0008 | 0.0006 |  | 0.1060 | 0.1384 | 0.1489 | 0.1172 |
| Laguna de Aguas Verdes | AVER | 37.04867 | -3.36838 | 3052 | 8 | 0.0006 | 0.0007 | 0.0008 | 0.0005 |  | 0.1128 | 0.1412 | 0.1521 | 0.1022 |
| Laguna Corral del Veleta | CVEL | 37.05864 | -3.36522 | 3081 | 8 | 0.0006 | 0.0007 | 0.0008 | 0.0005 |  | 0.1083 | 0.1372 | 0.1477 | 0.1051 |
| Laguna de Río Seco | RSEC | 37.05203 | -3.34570 | 3021 | 8 | 0.0006 | 0.0007 | 0.0008 | 0.0006 |  | 0.1115 | 0.1416 | 0.1526 | 0.1105 |
| Laguna de la Mosca | MOSC | 37.05976 | -3.31472 | 2893 | 8 | 0.0005 | 0.0007 | 0.0008 | 0.0005 |  | 0.1061 | 0.1342 | 0.1467 | 0.1026 |
| Laguna Hondera | HOND | 37.04794 | -3.29423 | 2892 | 8 | 0.0006 | 0.0007 | 0.0008 | 0.0006 |  | 0.1079 | 0.1388 | 0.1498 | 0.1118 |
| Laguna de Juntillas | JUNT | 37.11009 | -3.26432 | 2928 | 7 | 0.0005 | 0.0006 | 0.0007 | 0.0005 |  | 0.0995 | 0.1261 | 0.1422 | 0.0977 |
| (D) *Boreonectes ibericus* |  |  |  |  |  |  |  |  |  |  |  |  |  |  |
| Laguna Cuadrada | CUAD | 37.02707 | -3.41860 | 2913 | 8 | 0.0003 | 0.0004 | 0.0004 | 0.0004 |  | 0.0569 | 0.0771 | 0.0831 | 0.0706 |
| Laguna de Lanjarón | LLAN | 37.03772 | -3.40045 | 2981 | 8 | 0.0003 | 0.0004 | 0.0004 | 0.0003 |  | 0.0585 | 0.0742 | 0.0797 | 0.0617 |
| Lagunillas de la Virgen | VIRG | 37.05265 | -3.37886 | 2946 | 6 | 0.0003 | 0.0004 | 0.0005 | 0.0003 |  | 0.0624 | 0.0800 | 0.0910 | 0.0651 |
| Laguna de Aguas Verdes | AVER | 37.04867 | -3.36838 | 3052 | 8 | 0.0003 | 0.0004 | 0.0005 | 0.0004 |  | 0.0593 | 0.0815 | 0.0879 | 0.0785 |
| Laguna Larga | LARG | 37.05956 | -3.33441 | 2781 | 8 | 0.0003 | 0.0005 | 0.0005 | 0.0005 |  | 0.0627 | 0.0882 | 0.0951 | 0.0955 |
| Laguna de la Caldera | CALD | 37.05457 | -3.32921 | 3015 | 8 | 0.0003 | 0.0004 | 0.0005 | 0.0005 |  | 0.0548 | 0.0831 | 0.0903 | 0.1013 |
| Laguna Altera | ALTE | 37.05850 | -3.30446 | 3065 | 8 | 0.0003 | 0.0005 | 0.0005 | 0.0005 |  | 0.0623 | 0.0872 | 0.0940 | 0.0948 |
| Laguna del Peñón Negro | PNEG | 37.02872 | -3.29645 | 2830 | 8 | 0.0003 | 0.0004 | 0.0005 | 0.0004 |  | 0.0625 | 0.0840 | 0.0903 | 0.0829 |
| Laguna de Lavadero de la Reina | LAVR | 37.12385 | -3.27321 | 2633 | 8 | 0.0003 | 0.0005 | 0.0005 | 0.0005 |  | 0.0632 | 0.0866 | 0.0935 | 0.0873 |

*Note*: Average values across loci are presented for observed (*H*_O_) and expected (*H*_E_) heterozygosity, nucleotide diversity (π), and Wright's inbreeding coefficient (*F*_IS_). Genetic summary statistics were calculated in stacks for all positions (polymorphic and nonpolymorphic) and only variant positions (polymorphic) for populations with *n* ≥ 5 genotyped individuals (see Methods S2).

**TABLE S2** Attributes of genomic datasets obtained for each studied species of diving beetle in Sierra Nevada mountain range, including number of retained SNPs, number of retained reads after different quality filtering steps in stacks, coverage depth, and proportion of missing data.

|  |  | Number of reads | |  | Coverage depth | | | |  | | % of missing data | | | |
| --- | --- | --- | --- | --- | --- | --- | --- | --- | --- | --- | --- | --- | --- | --- |
| Species | SNPs | Mean | Range |  | Mean | Mode | Range |  | | Mean | | Mode | Range |  |
| *Agabus nevadensis* | 6282 | 1,936,978 | 147,011-3,890,200 |  | 38 | 22 | 9-62 |  | | 13 | | 8 | 6-59 |  |
| *Hydroporus marginatus* | 1985 | 1,049,707 | 306,024-1,979,189 |  | 26 | 42 | 12-44 |  | | 14 | | 9 | 7-32 |  |
| *Hydroporus sabaudus sierranevadensis* | 7206 | 1,895,478 | 248,098-3,562,270 |  | 35 | 30 | 15-62 |  | | 15 | | 6 | 6-58 |  |
| *Boreonectes ibericus* | 3416 | 1,458,933 | 304,906-2,179,285 |  | 23 | 25 | 9-32 |  | | 12 | | 10 | 6-44 |  |

**TABLE S3** Pairwise *F*_ST_ values (below the diagonal) and their corresponding *q*-values (above the diagonal) for populations of *Agabus nevadensis* from Sierra Nevada. Significance of pairwise *F*_ST_ values was determined with Fisher´s exact tests after 10,000 permutations, as implemented in arlequin v. 3.5. Statistically significant *F*_ST_ values after false discovery rate adjustment (FDR) to control for multiple tests (FDR of 5%, *q* < 0.05) are indicated in bold. Negative *F*_ST_ values are reported as zero. Pairwise *F*_ST_ values were calculated for populations with *n* ≥ 5 genotyped individuals. Population codes as described in Table S1.

| Code | CUAD | LLAN | VIRG | AVER | CALD | MOSC | HOND |
| --- | --- | --- | --- | --- | --- | --- | --- |
| CUAD | – | 0.000 | 0.001 | 0.001 | 0.001 | 0.001 | 0.002 |
| LLAN | **0.230** | – | 0.000 | 0.001 | 0.000 | 0.000 | 0.001 |
| VIRG | **0.230** | **0.073** | – | 0.001 | 0.001 | 0.001 | 0.002 |
| AVER | **0.234** | **0.097** | **0.044** | – | 0.000 | 0.000 | 0.001 |
| CALD | **0.212** | **0.140** | **0.132** | **0.108** | – | 0.001 | 0.002 |
| MOSC | **0.295** | **0.200** | **0.182** | **0.175** | **0.094** | – | 0.002 |
| HOND | **0.319** | **0.223** | **0.195** | **0.197** | **0.193** | **0.191** | – |

**TABLE S4** Pairwise *F*_ST_ values (below the diagonal) and their corresponding *q*-values (above the diagonal) for populations of *Hydroporus marginatus* from Sierra Nevada. Significance of pairwise *F*_ST_ values was determined with Fisher´s exact tests after 10,000 permutations, as implemented in arlequin v. 3.5. Statistically significant *F*_ST_ values after false discovery rate adjustment (FDR) to control for multiple tests (FDR of 5%, *q* < 0.05) are indicated in bold. Negative *F*_ST_ values are reported as zero. Population codes as described in Table S1.

| Code | CUAD | LLAN | AVER | LARG | CALD | MOSC | ALTE | BORR |
| --- | --- | --- | --- | --- | --- | --- | --- | --- |
| CUAD | – | 0.000 | 0.000 | 0.000 | 0.000 | 0.000 | 0.000 | 0.000 |
| LLAN | **0.136** | – | 0.000 | 0.000 | 0.000 | 0.000 | 0.000 | 0.000 |
| AVER | **0.182** | **0.143** | – | 0.000 | 0.000 | 0.000 | 0.000 | 0.000 |
| LARG | **0.209** | **0.234** | **0.184** | – | 0.000 | 0.000 | 0.000 | 0.000 |
| CALD | **0.220** | **0.225** | **0.157** | **0.131** | – | 0.000 | 0.000 | 0.000 |
| MOSC | **0.198** | **0.217** | **0.149** | **0.129** | **0.079** | – | 0.002 | 0.002 |
| ALTE | **0.275** | **0.268** | **0.196** | **0.176** | **0.108** | **0.049** | – | 0.920 |
| BORR | **0.274** | **0.276** | **0.198** | **0.190** | **0.119** | **0.055** | 0.000 | – |

**TABLE S5** Pairwise *F*_ST_ values (below the diagonal) and their corresponding *q*-values (above the diagonal) for populations of *Hydroporus sabaudus sierranevadensis* from Sierra Nevada. Significance of pairwise *F*_ST_ values was determined with Fisher´s exact tests after 10,000 permutations, as implemented in arlequin v. 3.5. None pairwise *F*_ST_ value was significantly different from zero after false discovery rate adjustment (FDR) to control for multiple tests (FDR of 5%, *q* < 0.05). Negative *F*_ST_ values are reported as zero. Population codes as described in Table S1.

| Code | CPAL | LILA | CERM | VIRG | AVER | CVEL | RSEC | MOSC | HOND | JUNT |
| --- | --- | --- | --- | --- | --- | --- | --- | --- | --- | --- |
| CPAL | – | 0.999 | 0.999 | 0.999 | 0.999 | 0.999 | 0.999 | 0.999 | 0.999 | 0.999 |
| LILA | 0.000 | – | 0.999 | 0.999 | 0.999 | 0.999 | 0.999 | 0.999 | 0.999 | 0.999 |
| CERM | 0.002 | 0.010 | – | 0.999 | 0.999 | 0.999 | 0.999 | 0.999 | 0.999 | 0.999 |
| VIRG | 0.000 | 0.014 | 0.010 | – | 0.999 | 0.999 | 0.999 | 0.999 | 0.999 | 0.999 |
| AVER | 0.000 | 0.011 | 0.009 | 0.013 | – | 0.999 | 0.999 | 0.999 | 0.999 | 0.999 |
| CVEL | 0.000 | 0.014 | 0.013 | 0.012 | 0.014 | – | 0.999 | 0.999 | 0.999 | 0.999 |
| RSEC | 0.000 | 0.009 | 0.003 | 0.011 | 0.010 | 0.011 | – | 0.999 | 0.999 | 0.999 |
| MOSC | 0.006 | 0.000 | 0.017 | 0.000 | 0.000 | 0.000 | 0.000 | – | 0.999 | 0.999 |
| HOND | 0.000 | 0.015 | 0.012 | 0.011 | 0.013 | 0.011 | 0.010 | 0.000 | – | 0.999 |
| JUNT | 0.019 | 0.000 | 0.003 | 0.000 | 0.000 | 0.000 | 0.000 | 0.008 | 0.000 | – |

**TABLE S6** Pairwise *F*_ST_ values (below the diagonal) and their corresponding *q*-values (above the diagonal) for populations of *Boreonectes ibericus* from Sierra Nevada. Significance of pairwise *F*_ST_ values was determined with Fisher´s exact tests after 10,000 permutations, as implemented in arlequin v. 3.5. Statistically significant *F*_ST_ values after false discovery rate adjustment (FDR) to control for multiple tests (FDR of 5%, *q* < 0.05) are indicated in bold. Negative *F*_ST_ values are reported as zero. Population codes as described in Table S1.

| Code | CUAD | LLAN | VIRG | AVER | LARG | CALD | ALTE | PNEG | LAVR |
| --- | --- | --- | --- | --- | --- | --- | --- | --- | --- |
| CUAD | – | 0.193 | 0.992 | 0.613 | 0.057 | 0.090 | 0.175 | 0.078 | 0.005 |
| LLAN | 0.030 | – | 0.505 | 0.175 | 0.012 | 0.029 | 0.029 | 0.012 | 0.005 |
| VIRG | 0.000 | 0.023 | – | 0.992 | 0.992 | 0.934 | 0.992 | 0.992 | 0.992 |
| AVER | 0.019 | 0.040 | 0.000 | – | 0.444 | 0.928 | 0.928 | 0.361 | 0.029 |
| LARG | 0.044 | **0.066** | 0.000 | 0.024 | – | 0.999 | 0.992 | 0.992 | 0.520 |
| CALD | 0.046 | **0.070** | 0.014 | 0.013 | 0.000 | – | 0.992 | 0.928 | 0.502 |
| ALTE | 0.033 | **0.051** | 0.000 | 0.014 | 0.007 | 0.006 | – | 0.992 | 0.175 |
| PNEG | 0.042 | **0.061** | 0.000 | 0.025 | 0.010 | 0.015 | 0.010 | – | 0.039 |
| LAVR | **0.071** | **0.106** | 0.008 | **0.044** | 0.021 | 0.022 | 0.028 | **0.035** | – |

**TABLE S7** Multiple matrix regressions with randomization (MMRR) for genetic differentiation (*F*_ST_) between populations in relation to weighted topographic distance and elevation dissimilarity. *R*^2^, coefficient of determination; β, standardized regression coefficient; *t*, *t*-statistic; *p*, one-tailed significance level

| Variable | β | *t* | *p* | |
| --- | --- | --- | --- | --- |
| (A) *Agabus nevadensis* (*R*^2^ = 0.498) | |  |  | |
| *Explanatory terms* |  |  |  | |
| Constant |  | 0.68 | 1.000 | |
| Weighted topographic distance | 0.676 | 4.34 | <0.001 | |
| *Rejected terms* |  |  |  | |
| Elevation dissimilarity |  | -1.01 | 0.798 | |
| (B) *Hydroporus marginatus* (*R*^2^ = 0.652) | |  |  | |
| *Explanatory terms* |  |  |  | |
| Constant |  | 0.70 | 0.999 | |
| Weighted topographic distance | 6.987 | 6.99 | <0.001 | |
| *Rejected terms* |  |  |  | |
| Elevation dissimilarity |  | 0.672 | 0.274 | |
| (C) *Hydroporus sabaudus sierranevadensis* (*R*^2^ = 0.000) | | | |  |
| *Rejected terms* |  |  |  | |
| Weighted topographic distance |  | -1.03 | 0.814 | |
| Elevation dissimilarity |  | -0.20 | 0.583 | |
| (D) *Boreonectes ibericus* (*R*^2^ = 0.365) | |  |  | |
| *Explanatory terms* |  |  |  | |
| Constant |  | -0.21 | 0.904 | |
| Weighted topographic distance | 0.680 | 4.43 | 0.003 | |
| *Rejected terms* |  |  |  | |
| Elevation dissimilarity |  | -0.39 | 0.614 | |

**TABLE S8** Model selection to assess the relationship between genetic diversity (nucleotide diversity, π) and geographical peripherality of populations (A), elevation (B), and pond area (C). Best ranked equivalent models (ΔAICc ≤ 2) are indicated in bold. *K*, number of parameters in the model; *R*^2^, coefficient of determination; AICc, corrected Akaike’s information criterion (AIC) value; ΔAICc, difference in AICc value from that of the strongest model; ω*_i_*, AICc weight.

| Model |  | *K* | *R*^2^ | AICc | ΔAICc | ω*_i_* |
| --- | --- | --- | --- | --- | --- | --- |
| (A) *Agabus nevadensis* | |  |  |  |  |  |
| **Null model** | | **0** | **0.000** | **-31.70** | **0.01** | **0.47** |
| **A** | | **1** | **0.633** | **-31.70** | **0.00** | **0.47** |
| B | | 1 | 0.310 | -27.30 | 4.42 | 0.05 |
| C | | 1 | 0.000 | -24.70 | 7.01 | 0.01 |
| A+B | | 2 | 0.640 | -17.80 | 13.86 | 0.00 |
| A+C | | 2 | 0.651 | -18.10 | 13.64 | 0.00 |
| B+C | | 2 | 0.337 | -13.60 | 18.14 | 0.00 |
| A+B+C | | 3 | 0.655 | 23.90 | 55.57 | 0.00 |
| (B) *Hydroporus marginatus* | |  |  |  |  |  |
| **Null model** | | **0** | **0.000** | **-44.70** | **0.00** | **0.74** |
| A | | 1 | 0.174 | -40.60 | 4.07 | 0.10 |
| B | | 1 | 0.158 | -40.50 | 4.23 | 0.09 |
| C | | 1 | 0.092 | -39.90 | 4.83 | 0.07 |
| A+B | | 2 | 0.338 | -33.10 | 11.63 | 0.00 |
| A+C | | 2 | 0.180 | -31.40 | 13.35 | 0.00 |
| B+C | | 2 | 0.187 | -31.40 | 13.28 | 0.00 |
| A+B+C | | 3 | 0.355 | -14.60 | 30.09 | 0.00 |
| (C) *Hydroporus sabaudus sierranevadensis* | |  |  |  |  |  |
| **Null model** | | **0** | **0.000** | **-82.00** | **1.08** | **0.29** |
| **A** | | **1** | **0.416** | **-83.10** | **0.00** | **0.50** |
| B | | 1 | 0.146 | -79.30 | 3.79 | 0.08 |
| C | | 1 | 0.093 | -78.70 | 4.40 | 0.06 |
| A+B | | 2 | 0.458 | -77.90 | 5.24 | 0.04 |
| A+C | | 2 | 0.426 | -77.30 | 5.82 | 0.03 |
| B+C | | 2 | 0.276 | -75.00 | 8.14 | 0.01 |
| A+B+C | | 3 | 0.459 | -68.90 | 14.23 | 0.00 |
| (D) *Boreonectes ibericus* |  |  |  |  |  |  |
| **Null model** |  | **0** | **0.000** | **-64.20** | **0.00** | **0.39** |
| **A** |  | **1** | **0.355** | **-63.30** | **0.85** | **0.26** |
| B |  | 1 | 0.120 | -60.50 | 3.65 | 0.06 |
| C |  | 1 | 0.112 | -60.40 | 3.73 | 0.06 |
| A+B |  | **2** | **0.694** | **-62.80** | **1.34** | **0.20** |
| A+C |  | 2 | 0.371 | -56.30 | 7.83 | 0.01 |
| B+C |  | 2 | 0.226 | -54.50 | 9.70 | 0.00 |
| A+B+C |  | 3 | 0.847 | -57.00 | 7.13 | 0.01 |

**TABLE S9** Taxa collected in the ponds studied in Sierra Nevada Massif. Pond codes as described in Table S1.

| Group | Taxa | AVER | RSEC | CALD | CUAD | LLAN | LILA | CPAL | ALTE | BORR | HOND | PNEG | JUNT | LAVR | LARG | MOSC | CERM | MEDE | VIRG | CVEL |
| --- | --- | --- | --- | --- | --- | --- | --- | --- | --- | --- | --- | --- | --- | --- | --- | --- | --- | --- | --- | --- |
| Platyhelminthes | Planariidae | 0 | 0 | 0 | 0 | 0 | 1 | 0 | 0 | 1 | 1 | 0 | 0 | 0 | 0 | 1 | 1 | 1 | 1 | 0 |
| Mollusca | *Euglesa* sp. | 1 | 1 | 0 | 0 | 1 | 1 | 1 | 0 | 1 | 1 | 0 | 1 | 1 | 0 | 0 | 1 | 1 | 1 | 0 |
| Annelida | Lumbriculidae | 0 | 1 | 0 | 1 | 1 | 1 | 0 | 1 | 1 | 1 | 0 | 0 | 0 | 1 | 1 | 0 | 0 | 0 | 0 |
| Annelida | Glossiphoniidae | 0 | 0 | 0 | 0 | 0 | 0 | 0 | 0 | 0 | 0 | 0 | 0 | 1 | 0 | 0 | 0 | 0 | 0 | 0 |
| Diptera | Tanypodinae | 1 | 1 | 0 | 0 | 1 | 1 | 1 | 0 | 1 | 1 | 1 | 1 | 1 | 1 | 1 | 0 | 0 | 1 | 0 |
| Diptera | Chironominae | 1 | 1 | 1 | 1 | 1 | 1 | 1 | 1 | 1 | 1 | 0 | 1 | 0 | 1 | 0 | 0 | 1 | 0 | 1 |
| Diptera | Orthocladinae | 1 | 1 | 1 | 1 | 1 | 1 | 1 | 1 | 1 | 1 | 1 | 1 | 1 | 1 | 1 | 1 | 1 | 1 | 1 |
| Diptera | Diamesinae | 0 | 0 | 0 | 0 | 0 | 0 | 0 | 0 | 0 | 0 | 0 | 0 | 0 | 0 | 1 | 0 | 0 | 0 | 1 |
| Diptera | *Culex* sp. | 0 | 0 | 0 | 0 | 0 | 0 | 0 | 0 | 0 | 0 | 0 | 0 | 1 | 0 | 0 | 0 | 0 | 0 | 0 |
| Diptera | Tipulidae | 0 | 0 | 0 | 0 | 0 | 1 | 0 | 0 | 0 | 0 | 0 | 0 | 0 | 0 | 0 | 0 | 0 | 0 | 0 |
| Diptera | Tabanidae | 0 | 0 | 0 | 0 | 0 | 0 | 0 | 0 | 0 | 0 | 0 | 0 | 1 | 0 | 0 | 1 | 0 | 0 | 0 |
| Diptera | Ephydridae | 0 | 0 | 0 | 0 | 0 | 0 | 0 | 0 | 0 | 0 | 0 | 0 | 1 | 0 | 0 | 0 | 0 | 0 | 0 |
| Odonata | *Libellula depressa* | 0 | 0 | 0 | 0 | 0 | 0 | 0 | 0 | 0 | 0 | 0 | 0 | 1 | 0 | 0 | 0 | 0 | 0 | 0 |
| Odonata | *Sympetrum striolatum* | 0 | 0 | 0 | 0 | 0 | 0 | 0 | 0 | 0 | 0 | 0 | 0 | 1 | 0 | 0 | 0 | 0 | 0 | 0 |
| Odonata | *Pyrrhosoma nymphula* | 0 | 0 | 0 | 0 | 0 | 0 | 0 | 0 | 0 | 0 | 0 | 0 | 1 | 0 | 0 | 0 | 0 | 0 | 0 |
| Odonata | *Anax* sp. | 0 | 0 | 0 | 0 | 0 | 0 | 0 | 0 | 0 | 0 | 0 | 0 | 0 | 0 | 0 | 0 | 0 | 0 | 0 |
| Coleoptera | *Agabus nevadensis* | 1 | 1 | 1 | 1 | 1 | 1 | 1 | 1 | 1 | 1 | 1 | 1 | 1 | 1 | 1 | 1 | 1 | 1 | 1 |
| Coleoptera | *Agabus nebulosus* | 0 | 0 | 0 | 0 | 0 | 0 | 0 | 0 | 1 | 0 | 0 | 1 | 1 | 0 | 0 | 1 | 0 | 1 | 0 |
| Coleoptera | *Agabus biguttatus* | 0 | 0 | 0 | 0 | 0 | 0 | 0 | 0 | 0 | 0 | 0 | 0 | 0 | 0 | 0 | 0 | 0 | 0 | 0 |
| Coleoptera | *Boreonectes ibericus* | 1 | 1 | 1 | 1 | 1 | 1 | 1 | 1 | 1 | 1 | 1 | 1 | 1 | 1 | 1 | 1 | 1 | 1 | 0 |
| Coleoptera | *Hydroporus sabaudus sierranevadensis* | 1 | 1 | 1 | 1 | 1 | 1 | 1 | 1 | 1 | 1 | 1 | 1 | 0 | 1 | 1 | 1 | 1 | 1 | 1 |
| Coleoptera | *Hydroporus marginatus* | 1 | 1 | 1 | 1 | 1 | 1 | 0 | 1 | 1 | 0 | 1 | 0 | 0 | 1 | 1 | 0 | 0 | 0 | 0 |
| Coleoptera | *Hydroporus nevadensis* | 1 | 1 | 0 | 1 | 1 | 1 | 0 | 0 | 1 | 1 | 0 | 1 | 1 | 0 | 1 | 0 | 0 | 0 | 1 |
| Coleoptera | *Hydroporus nigrita* | 1 | 0 | 1 | 1 | 0 | 0 | 0 | 0 | 1 | 1 | 0 | 1 | 0 | 0 | 0 | 0 | 0 | 0 | 1 |
| Coleoptera | *Hydroporus normandi alhambrae* | 0 | 0 | 0 | 0 | 0 | 0 | 0 | 0 | 0 | 0 | 0 | 0 | 0 | 0 | 0 | 1 | 0 | 0 | 0 |
| Coleoptera | *Limnohydrobius convexus* | 0 | 0 | 0 | 0 | 0 | 0 | 0 | 0 | 0 | 0 | 0 | 0 | 1 | 0 | 0 | 0 | 0 | 0 | 0 |
| Coleoptera | *Helophorus glacialis* | 1 | 1 | 1 | 0 | 1 | 1 | 1 | 1 | 1 | 1 | 1 | 1 | 1 | 1 | 1 | 1 | 1 | 1 | 1 |
| Coleoptera | *Helophorus nevadensis* | 1 | 0 | 1 | 1 | 0 | 1 | 0 | 0 | 0 | 1 | 0 | 0 | 0 | 0 | 1 | 0 | 0 | 1 | 1 |
| Coleoptera | *Enochrus fuscipennis* | 1 | 1 | 0 | 1 | 1 | 0 | 0 | 0 | 1 | 1 | 0 | 1 | 1 | 0 | 1 | 0 | 0 | 1 | 0 |
| Coleoptera | *Limnebius truncatellus* | 1 | 1 | 1 | 1 | 0 | 1 | 0 | 1 | 1 | 1 | 1 | 0 | 1 | 0 | 0 | 0 | 1 | 0 | 1 |
| Coleoptera | *Ochthebius quadrifoveolatus* | 0 | 1 | 0 | 0 | 0 | 0 | 0 | 1 | 0 | 0 | 0 | 0 | 0 | 0 | 0 | 0 | 0 | 0 | 0 |
| Coleoptera | *Ochthebius semotus* | 0 | 0 | 0 | 0 | 0 | 0 | 0 | 0 | 0 | 0 | 0 | 0 | 0 | 0 | 0 | 0 | 0 | 0 | 0 |
| Coleoptera | *Limnius opacus* | 0 | 0 | 1 | 0 | 1 | 0 | 0 | 1 | 0 | 0 | 0 | 0 | 0 | 0 | 0 | 0 | 0 | 0 | 0 |
| Coleoptera | *Laccobius obscuratus* | 0 | 0 | 1 | 0 | 0 | 0 | 0 | 0 | 0 | 0 | 1 | 0 | 0 | 0 | 0 | 0 | 0 | 0 | 0 |
| Coleoptera | *Elmis sp.* | 0 | 0 | 0 | 0 | 0 | 0 | 0 | 0 | 0 | 0 | 0 | 0 | 0 | 0 | 1 | 0 | 0 | 0 | 0 |
| Coleoptera | *Laccophilus minutus* | 0 | 0 | 0 | 0 | 0 | 0 | 0 | 0 | 0 | 0 | 0 | 0 | 0 | 0 | 1 | 0 | 0 | 0 | 0 |
| Coleoptera | *Laccophilus hyalinus* | 0 | 0 | 0 | 0 | 0 | 0 | 0 | 0 | 0 | 0 | 0 | 0 | 0 | 0 | 0 | 0 | 0 | 0 | 0 |
| Coleoptera | *Hydroglyphus geminus* | 0 | 0 | 0 | 0 | 0 | 0 | 0 | 0 | 0 | 0 | 0 | 1 | 0 | 0 | 0 | 0 | 0 | 0 | 0 |
| Coleoptera | *Haliplus lineatocollis* | 0 | 0 | 0 | 0 | 0 | 0 | 0 | 0 | 1 | 0 | 1 | 0 | 0 | 0 | 0 | 0 | 0 | 0 | 0 |
| Hemiptera | *Nepa cinerea* | 0 | 0 | 0 | 0 | 0 | 0 | 0 | 0 | 0 | 0 | 0 | 0 | 1 | 0 | 0 | 0 | 0 | 0 | 0 |
| Hemiptera | *Hydrometra stagnorum* | 0 | 0 | 0 | 0 | 0 | 0 | 0 | 0 | 0 | 0 | 0 | 0 | 1 | 0 | 0 | 0 | 0 | 0 | 0 |
| Hemiptera | *Corixa affinis* | 0 | 0 | 0 | 0 | 0 | 0 | 0 | 0 | 0 | 0 | 1 | 0 | 0 | 0 | 0 | 0 | 0 | 0 | 0 |
| Hemiptera | *Micronecta poweri* | 0 | 0 | 0 | 0 | 0 | 0 | 0 | 0 | 0 | 0 | 0 | 0 | 0 | 0 | 1 | 0 | 0 | 0 | 0 |
| Hemiptera | *Notonecta meridionalis* | 0 | 0 | 0 | 0 | 0 | 0 | 0 | 0 | 0 | 0 | 0 | 0 | 0 | 0 | 0 | 0 | 0 | 0 | 0 |
| Hemiptera | *Notonecta glauca* | 0 | 0 | 0 | 0 | 0 | 1 | 0 | 0 | 0 | 0 | 0 | 1 | 1 | 0 | 1 | 0 | 0 | 0 | 0 |
| Hemiptera | *Notonecta maculata* | 0 | 0 | 0 | 0 | 0 | 1 | 0 | 1 | 0 | 0 | 0 | 0 | 0 | 0 | 0 | 0 | 0 | 0 | 0 |
| Hemiptera | *Paracorixa concinna* | 0 | 0 | 0 | 0 | 0 | 0 | 0 | 1 | 0 | 0 | 0 | 0 | 0 | 0 | 0 | 0 | 0 | 0 | 0 |
| Hemiptera | *Sigara nigrolineata* | 0 | 1 | 0 | 0 | 0 | 0 | 1 | 0 | 0 | 0 | 1 | 1 | 1 | 0 | 0 | 0 | 0 | 0 | 0 |
| Hemiptera | *Gerris costai poissoni* | 0 | 0 | 0 | 0 | 0 | 0 | 0 | 0 | 0 | 0 | 0 | 0 | 0 | 0 | 0 | 0 | 1 | 0 | 0 |
| Hemiptera | *Gerris gibbifer* | 0 | 0 | 0 | 0 | 0 | 0 | 0 | 0 | 0 | 0 | 0 | 0 | 1 | 0 | 0 | 0 | 0 | 0 | 0 |
| Hemiptera | *Gerris thoracicus* | 1 | 0 | 0 | 0 | 0 | 0 | 0 | 0 | 0 | 0 | 0 | 1 | 1 | 0 | 0 | 0 | 0 | 0 | 0 |
| Hemiptera | *Arctocorixa carinata* | 1 | 0 | 0 | 0 | 0 | 0 | 0 | 0 | 0 | 0 | 0 | 0 | 0 | 0 | 0 | 0 | 0 | 0 | 0 |
| Ephemeroptera | *Cloëon* sp. | 0 | 0 | 0 | 0 | 0 | 0 | 0 | 0 | 0 | 0 | 1 | 0 | 1 | 0 | 0 | 0 | 0 | 0 | 0 |
| Plecoptera | Leuctridae | 0 | 0 | 0 | 0 | 0 | 0 | 0 | 0 | 0 | 0 | 0 | 0 | 0 | 0 | 0 | 0 | 1 | 0 | 0 |
| Trichoptera | Limnephilidae | 0 | 0 | 0 | 0 | 0 | 1 | 0 | 0 | 0 | 0 | 0 | 0 | 0 | 0 | 0 | 0 | 0 | 1 | 0 |
| Trichoptera | Policentropodidae | 0 | 0 | 0 | 0 | 0 | 0 | 0 | 0 | 0 | 0 | 0 | 0 | 0 | 0 | 0 | 0 | 0 | 0 | 0 |
| Arachnida | Hydrachnidia | 0 | 0 | 0 | 0 | 0 | 1 | 0 | 0 | 1 | 0 | 0 | 0 | 0 | 0 | 0 | 0 | 1 | 0 | 0 |
| Crustacea | Ostracoda | 1 | 0 | 0 | 0 | 0 | 0 | 0 | 0 | 0 | 0 | 0 | 0 | 0 | 0 | 0 | 0 | 0 | 0 | 0 |
| Crustacea | Chydoridae | 0 | 0 | 0 | 1 | 0 | 0 | 0 | 0 | 1 | 0 | 0 | 0 | 0 | 0 | 0 | 0 | 0 | 0 | 0 |
| Crustacea | Diaptomidae | 0 | 0 | 0 | 0 | 1 | 1 | 1 | 1 | 0 | 0 | 1 | 0 | 0 | 0 | 1 | 0 | 0 | 0 | 0 |
| Crustacea | Cyclopidae | 0 | 0 | 0 | 0 | 0 | 0 | 0 | 0 | 0 | 0 | 0 | 0 | 0 | 0 | 0 | 0 | 0 | 0 | 0 |

**TABLE S10** Pairwise beta diversity among ponds computed as Sørensen’s dissimilarity. Population codes as described in Table S1.

| Code | AVER | RSEC | CALD | CUAD | LLAN | LILA | CPAL | ALTE | BORR | HOND | PNEG | JUNT | LAVR | LARG | MOSC | CERM | MEDE | VIRG |
| --- | --- | --- | --- | --- | --- | --- | --- | --- | --- | --- | --- | --- | --- | --- | --- | --- | --- | --- |
| RSEC | 0.273 |  |  |  |  |  |  |  |  |  |  |  |  |  |  |  |  |  |
| CALD | 0.290 | 0.357 |  |  |  |  |  |  |  |  |  |  |  |  |  |  |  |  |
| CUAD | 0.250 | 0.310 | 0.259 |  |  |  |  |  |  |  |  |  |  |  |  |  |  |  |
| LLAN | 0.313 | 0.172 | 0.333 | 0.357 |  |  |  |  |  |  |  |  |  |  |  |  |  |  |
| LILA | 0.368 | 0.314 | 0.394 | 0.412 | 0.294 |  |  |  |  |  |  |  |  |  |  |  |  |  |
| CPAL | 0.429 | 0.280 | 0.478 | 0.583 | 0.250 | 0.400 |  |  |  |  |  |  |  |  |  |  |  |  |
| ALTE | 0.500 | 0.310 | 0.333 | 0.429 | 0.286 | 0.353 | 0.417 |  |  |  |  |  |  |  |  |  |  |  |
| BORR | 0.263 | 0.257 | 0.394 | 0.235 | 0.294 | 0.300 | 0.467 | 0.471 |  |  |  |  |  |  |  |  |  |  |
| HOND | 0.212 | 0.200 | 0.286 | 0.241 | 0.241 | 0.257 | 0.360 | 0.448 | 0.200 |  |  |  |  |  |  |  |  |  |
| PNEG | 0.500 | 0.379 | 0.407 | 0.571 | 0.429 | 0.471 | 0.333 | 0.429 | 0.471 | 0.517 |  |  |  |  |  |  |  |  |
| JUNT | 0.294 | 0.290 | 0.448 | 0.467 | 0.333 | 0.444 | 0.308 | 0.600 | 0.333 | 0.290 | 0.533 |  |  |  |  |  |  |  |
| LAVR | 0.511 | 0.524 | 0.700 | 0.659 | 0.610 | 0.617 | 0.622 | 0.756 | 0.532 | 0.571 | 0.610 | 0.442 |  |  |  |  |  |  |
| LARG | 0.407 | 0.250 | 0.364 | 0.391 | 0.217 | 0.379 | 0.263 | 0.304 | 0.379 | 0.333 | 0.391 | 0.440 | 0.722 |  |  |  |  |  |
| MOSC | 0.405 | 0.412 | 0.500 | 0.394 | 0.333 | 0.333 | 0.517 | 0.515 | 0.385 | 0.353 | 0.515 | 0.486 | 0.609 | 0.429 |  |  |  |  |
| CERM | 0.467 | 0.407 | 0.440 | 0.538 | 0.462 | 0.375 | 0.364 | 0.462 | 0.375 | 0.333 | 0.538 | 0.500 | 0.692 | 0.429 | 0.613 |  |  |  |
| MEDE | 0.429 | 0.360 | 0.565 | 0.583 | 0.333 | 0.467 | 0.300 | 0.583 | 0.400 | 0.280 | 0.583 | 0.385 | 0.676 | 0.368 | 0.517 | 0.364 |  |  |
| VIRG | 0.400 | 0.407 | 0.520 | 0.538 | 0.385 | 0.375 | 0.364 | 0.615 | 0.375 | 0.259 | 0.538 | 0.357 | 0.590 | 0.429 | 0.419 | 0.417 | 0.273 |  |
| CVEL | 0.357 | 0.440 | 0.217 | 0.333 | 0.500 | 0.467 | 0.500 | 0.500 | 0.467 | 0.280 | 0.583 | 0.462 | 0.730 | 0.474 | 0.517 | 0.455 | 0.600 | 0.545 |

**TABLE S11** Pairwise beta diversity among ponds computed as Simpson´s dissimilarity. Population codes as described in Table S1.

| Code | AVER | RSEC | CALD | CUAD | LLAN | LILA | CPAL | ALTE | BORR | HOND | PNEG | JUNT | LAVR | LARG | MOSC | CERM | MEDE | VIRG |
| --- | --- | --- | --- | --- | --- | --- | --- | --- | --- | --- | --- | --- | --- | --- | --- | --- | --- | --- |
| RSEC | 0.200 |  |  |  |  |  |  |  |  |  |  |  |  |  |  |  |  |  |
| CALD | 0.154 | 0.308 |  |  |  |  |  |  |  |  |  |  |  |  |  |  |  |  |
| CUAD | 0.143 | 0.286 | 0.231 |  |  |  |  |  |  |  |  |  |  |  |  |  |  |  |
| LLAN | 0.214 | 0.143 | 0.308 | 0.357 |  |  |  |  |  |  |  |  |  |  |  |  |  |  |
| LILA | 0.333 | 0.200 | 0.231 | 0.286 | 0.143 |  |  |  |  |  |  |  |  |  |  |  |  |  |
| CPAL | 0.200 | 0.100 | 0.400 | 0.500 | 0.100 | 0.100 |  |  |  |  |  |  |  |  |  |  |  |  |
| ALTE | 0.429 | 0.286 | 0.308 | 0.429 | 0.286 | 0.214 | 0.300 |  |  |  |  |  |  |  |  |  |  |  |
| BORR | 0.222 | 0.133 | 0.231 | 0.071 | 0.143 | 0.300 | 0.200 | 0.357 |  |  |  |  |  |  |  |  |  |  |
| HOND | 0.133 | 0.200 | 0.231 | 0.214 | 0.214 | 0.133 | 0.200 | 0.429 | 0.067 |  |  |  |  |  |  |  |  |  |
| PNEG | 0.429 | 0.357 | 0.385 | 0.571 | 0.429 | 0.357 | 0.200 | 0.429 | 0.357 | 0.500 |  |  |  |  |  |  |  |  |
| JUNT | 0.250 | 0.267 | 0.385 | 0.429 | 0.286 | 0.375 | 0.100 | 0.571 | 0.250 | 0.267 | 0.500 |  |  |  |  |  |  |  |
| LAVR | 0.389 | 0.333 | 0.538 | 0.500 | 0.429 | 0.550 | 0.300 | 0.643 | 0.450 | 0.400 | 0.429 | 0.250 |  |  |  |  |  |  |
| LARG | 0.111 | 0.000 | 0.222 | 0.222 | 0.000 | 0.000 | 0.222 | 0.111 | 0.000 | 0.111 | 0.222 | 0.222 | 0.444 |  |  |  |  |  |
| MOSC | 0.389 | 0.333 | 0.385 | 0.286 | 0.214 | 0.316 | 0.300 | 0.429 | 0.368 | 0.267 | 0.429 | 0.438 | 0.526 | 0.111 |  |  |  |  |
| CERM | 0.333 | 0.333 | 0.417 | 0.500 | 0.417 | 0.167 | 0.300 | 0.417 | 0.167 | 0.250 | 0.500 | 0.417 | 0.500 | 0.333 | 0.500 |  |  |  |
| MEDE | 0.200 | 0.200 | 0.500 | 0.500 | 0.200 | 0.200 | 0.300 | 0.500 | 0.100 | 0.100 | 0.500 | 0.200 | 0.400 | 0.333 | 0.300 | 0.300 |  |  |
| VIRG | 0.250 | 0.333 | 0.500 | 0.500 | 0.333 | 0.167 | 0.300 | 0.583 | 0.167 | 0.167 | 0.500 | 0.250 | 0.333 | 0.333 | 0.250 | 0.417 | 0.200 |  |
| CVEL | 0.100 | 0.300 | 0.100 | 0.200 | 0.400 | 0.200 | 0.500 | 0.400 | 0.200 | 0.100 | 0.500 | 0.300 | 0.500 | 0.444 | 0.300 | 0.400 | 0.600 | 0.500 |

**TABLE S12** Pairwise beta diversity among ponds computed as nestedness. Population codes as described in Table S1.

| Code | AVER | RSEC | CALD | CUAD | LLAN | LILA | CPAL | ALTE | BORR | HOND | PNEG | JUNT | LAVR | LARG | MOSC | CERM | MEDE | VIRG |
| --- | --- | --- | --- | --- | --- | --- | --- | --- | --- | --- | --- | --- | --- | --- | --- | --- | --- | --- |
| RSEC | 0.073 |  |  |  |  |  |  |  |  |  |  |  |  |  |  |  |  |  |
| CALD | 0.136 | 0.049 |  |  |  |  |  |  |  |  |  |  |  |  |  |  |  |  |
| CUAD | 0.107 | 0.025 | 0.028 |  |  |  |  |  |  |  |  |  |  |  |  |  |  |  |
| LLAN | 0.098 | 0.030 | 0.026 | 0.000 |  |  |  |  |  |  |  |  |  |  |  |  |  |  |
| LILA | 0.035 | 0.114 | 0.163 | 0.126 | 0.151 |  |  |  |  |  |  |  |  |  |  |  |  |  |
| CPAL | 0.229 | 0.180 | 0.078 | 0.083 | 0.150 | 0.300 |  |  |  |  |  |  |  |  |  |  |  |  |
| ALTE | 0.071 | 0.025 | 0.026 | 0.000 | 0.000 | 0.139 | 0.117 |  |  |  |  |  |  |  |  |  |  |  |
| BORR | 0.041 | 0.124 | 0.163 | 0.164 | 0.151 | 0.000 | 0.267 | 0.113 |  |  |  |  |  |  |  |  |  |  |
| HOND | 0.079 | 0.000 | 0.055 | 0.027 | 0.027 | 0.124 | 0.160 | 0.020 | 0.133 |  |  |  |  |  |  |  |  |  |
| PNEG | 0.071 | 0.022 | 0.023 | 0.000 | 0.000 | 0.113 | 0.133 | 0.000 | 0.113 | 0.017 |  |  |  |  |  |  |  |  |
| JUNT | 0.044 | 0.024 | 0.064 | 0.038 | 0.048 | 0.069 | 0.208 | 0.029 | 0.083 | 0.024 | 0.033 |  |  |  |  |  |  |  |
| LAVR | 0.122 | 0.190 | 0.162 | 0.159 | 0.181 | 0.067 | 0.322 | 0.113 | 0.082 | 0.171 | 0.181 | 0.192 |  |  |  |  |  |  |
| LARG | 0.296 | 0.250 | 0.141 | 0.169 | 0.217 | 0.379 | 0.041 | 0.193 | 0.379 | 0.222 | 0.169 | 0.218 | 0.278 |  |  |  |  |  |
| MOSC | 0.017 | 0.078 | 0.115 | 0.108 | 0.119 | 0.018 | 0.217 | 0.087 | 0.016 | 0.086 | 0.087 | 0.048 | 0.082 | 0.317 |  |  |  |  |
| CERM | 0.133 | 0.074 | 0.023 | 0.038 | 0.045 | 0.208 | 0.064 | 0.045 | 0.208 | 0.083 | 0.038 | 0.083 | 0.192 | 0.095 | 0.113 |  |  |  |
| MEDE | 0.229 | 0.160 | 0.065 | 0.083 | 0.133 | 0.267 | 0.000 | 0.083 | 0.300 | 0.180 | 0.083 | 0.185 | 0.276 | 0.035 | 0.217 | 0.064 |  |  |
| VIRG | 0.150 | 0.074 | 0.020 | 0.038 | 0.051 | 0.208 | 0.064 | 0.032 | 0.208 | 0.093 | 0.038 | 0.107 | 0.256 | 0.095 | 0.169 | 0.000 | 0.073 |  |
| CVEL | 0.257 | 0.140 | 0.117 | 0.133 | 0.100 | 0.267 | 0.000 | 0.100 | 0.267 | 0.180 | 0.083 | 0.162 | 0.230 | 0.029 | 0.217 | 0.055 | 0.000 | 0.045 |

**TABLE S13** Pearson correlations between population genetic diversity (π) of each focal taxon and species richness (α-diversity) of the local communities. *r*, Pearson correlation coefficient; *p*, significance level; *n*, number of analyzed populations.

| Species |  | *r* | *p* | *n* |
| --- | --- | --- | --- | --- |
| (A) *Agabus nevadensis* | | -0.123 | 0.771 | 7 |
| (B) *Hydroporus marginatus* | | -0.276 | 0.507 | 8 |
| (C) *Hydroporus sabaudus sierranevadensis* | | 0.247 | 0.492 | 10 |
| (D) *Boreonectes ibericus* |  | 0.068 | 0.863 | 9 |

**TABLE S14** Mantel tests assessing the correlation between genetic differentiation among populations of each taxon and community dissimilarity (β-diversity), estimated using Sørensen’s dissimilarity, Simpson’s dissimilarity, or the nestedness component. *r*, Mantel correlation coefficient; *p*, one-tailed significance level; *n*, number of analyzed populations.

| Variable |  | *r* | *p* | *n* |
| --- | --- | --- | --- | --- |
| (A) *Agabus nevadensis* | |  |  |  |
| Sørensen’s dissimilarity | | 0.409 | 0.883 | 7 |
| Simpson´s dissimilarity | | 0.145 | 0.735 | 7 |
| Nestedness component | | 0.402 | 0.895 | 7 |
| (B) *Hydroporus marginatus* | |  |  |  |
| Sørensen’s dissimilarity | | 0.566 | 0.997 | 8 |
| Simpson´s dissimilarity | | 0.390 | 0.967 | 8 |
| Nestedness component | | 0.026 | 0.471 | 8 |
| (C) *Hydroporus sabaudus sierranevadensis* | |  |  |  |
| Sørensen’s dissimilarity | | 0.032 | 0.574 | 10 |
| Simpson´s dissimilarity | | 0.164 | 0.823 | 10 |
| Nestedness component | | 0.202 | 0.075 | 10 |
| (D) *Boreonectes ibericus* |  |  |  |  |
| Sørensen’s dissimilarity |  | 0.076 | 0.383 | 9 |
| Simpson´s dissimilarity |  | 0.107 | 0.345 | 9 |
| Nestedness component |  | 0.050 | 0.564 | 9 |

**Supplementary figures**

**figure S1** Results of structure for (A) *Agabus nevadensis*, (B) *Hydroporus marginatus*, (C) *Hydroporus sabaudus sierranevadensis*, and (D) *Boreonectes ibericus*. Panels show mean (±SD) log probability of the data (LnPr(X|*K*)) over 10 runs of structure (left axes, black dots and error bars) for each value of *K* and the magnitude of Δ*K* (right axes, open blue triangles).

**FIGURE S2** Results of genetic assignments based on the program structure for *Agabus nevadensis*. Each individual is represented by a vertical bar partitioned into *K* coloured segments showing the individual’s probability of belonging to the cluster with that colour. Vertical black lines separate individuals from different populations. Population codes as described in Table S1.

**FIGURE S3** Results of genetic assignments based on the program structure for *Hydroporus marginatus*. Each individual is represented by a vertical bar partitioned into *K* coloured segments showing the individual’s probability of belonging to the cluster with that colour. Vertical black lines separate individuals from different populations. Population codes as described in Table S1.

**FIGURE S4** Results of genetic assignments based on the program structure for *Hydroporus sabaudus sierranevadensis*. Each individual is represented by a vertical bar partitioned into *K* coloured segments showing the individual’s probability of belonging to the cluster with that colour. Vertical black lines separate individuals from different populations. Population codes as described in Table S1.

**FIGURE S5** Results of genetic assignments based on the program structure for *Boreonectes ibericus*. Each individual is represented by a vertical bar partitioned into *K* coloured segments showing the individual’s probability of belonging to the cluster with that colour. Vertical black lines separate individuals from different populations. Population codes as described in Table S1.

**FIGURE S6** (A) Elytron area and (B) wing loading (elytron area/wing area) for females (left) and males (right) of *Agabus nevadensis* (♀ = 24, ♂ = 17), *Hydroporus marginatus* (♀ = 15, ♂ = 15), *Hydroporus sabaudus sierranevadensis* (♀ = 16, ♂ = 14), and *Boreonectes ibericus* (♀ = 15, ♂ = 18). Violin plots show estimated values for each trait (small colored dots) and mean and confidence intervals (black dots and vertical bars, respectively). Different lowercase letters above the plots indicate statistically significant differences among taxa based on post hoc Bonferroni-corrected Dunn's tests (*p* < 0.05).


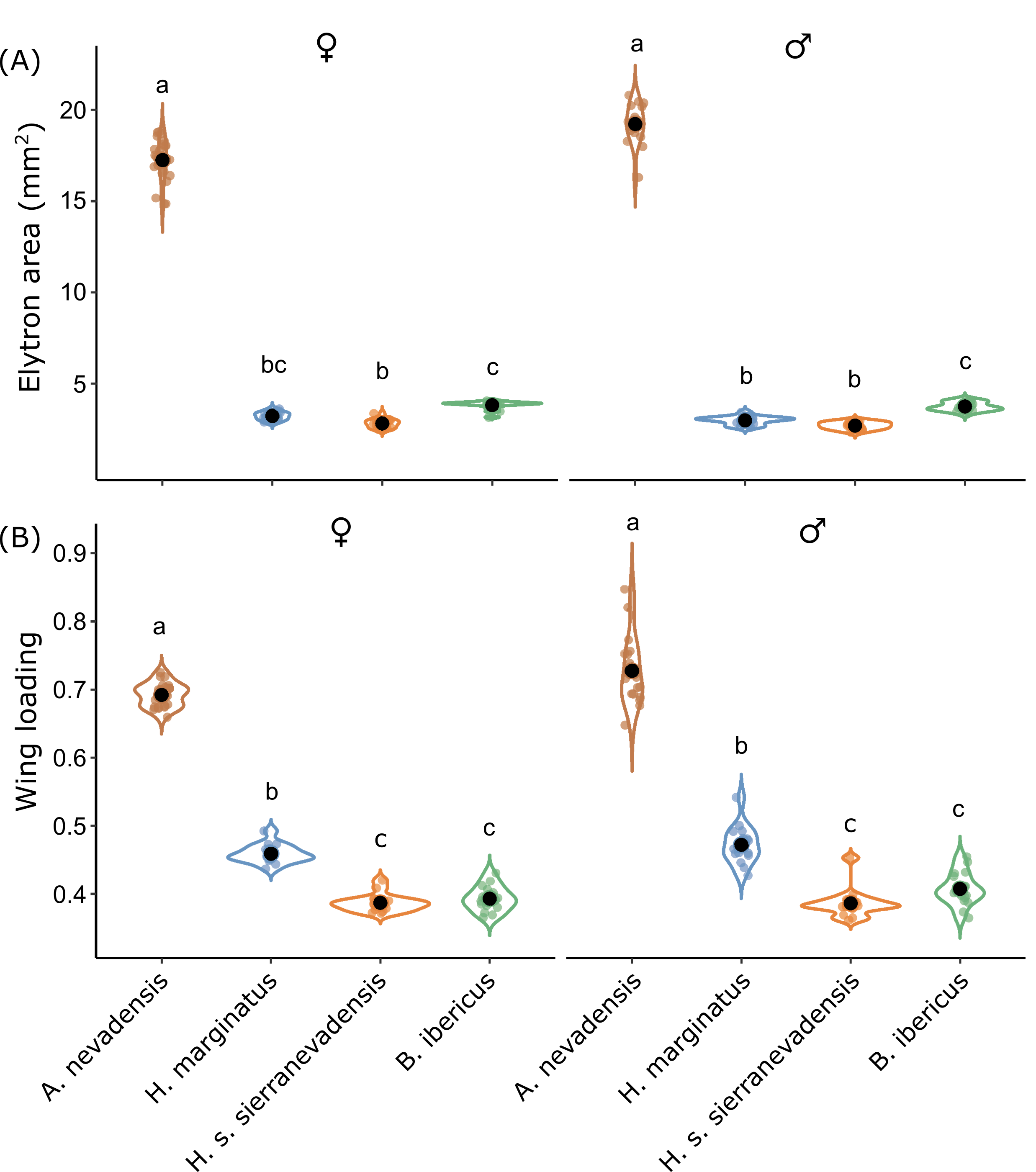


**References**

Bolger, A. M., Lohse, M., & Usadel, B. (2014). trimmomatic: a flexible trimmer for Illumina sequence data. *Bioinformatics, 30*(15), 2114-2120. doi:10.1093/bioinformatics/btu170

Catchen, J., Hohenlohe, P. A., Bassham, S., Amores, A., & Cresko, W. A. (2013). stacks: an analysis tool set for population genomics. *Molecular Ecology, 22*(11), 3124-3140. doi:10.1111/mec.12354

Danecek, P., Auton, A., Abecasis, G., Albers, C. A., Banks, E., DePristo, M. A., . . . Grp, G. P. A. (2011). The variant call format and vcftools. *Bioinformatics, 27*(15), 2156-2158. doi:10.1093/bioinformatics/btr330

Manichaikul, A., Mychaleckyj, J. C., Rich, S. S., Daly, K., Sale, M., & Chen, W. M. (2010). Robust relationship inference in genome-wide association studies. *Bioinformatics, 26*(22), 2867-2873. doi:10.1093/bioinformatics/btq559

Papadopoulou, A., & Knowles, L. L. (2015). Genomic tests of the species-pump hypothesis: Recent island connectivity cycles drive population divergence but not speciation in Caribbean crickets across the Virgin Islands. *Evolution, 69*(6), 1501-1517. doi:10.1111/evo.12667

Rochette, N. C., Rivera-Colón, A. G., & Catchen, J. M. (2019). stacks 2: Analytical methods for paired-end sequencing improve RADseq-based population genomics. *Molecular Ecology, 28*(21), 4737-4754. doi:10.1111/mec.15253
